# Skeletal Muscle Mitochondrial and Perilipin Content in a Cohort of Obese Subjects Undergoing Moderate and High Intensity Training

**DOI:** 10.3390/metabo12090855

**Published:** 2022-09-11

**Authors:** Giuseppe Sirago, Filippo Vaccari, Stefano Lazzer, Andrea D’Amuri, Juana M. Sanz, Marco V. Narici, Carlo Reggiani, Angelina Passaro, Luana Toniolo

**Affiliations:** 1 Department of Biomedical Sciences, University of Padova, 35131 Padova, Italy; 2 Department of Medicine, School of Sport Sciences, University of Udine, 33100 Udine, Italy; 3 Department of Translational Medicine, University of Ferrara, 44121 Ferrara, Italy; 4 Department of Chemical, Pharmaceutical and Agricultural Sciences, University of Ferrara, Via Luigi Borsari 46, 44121 Ferrara, Italy; 5 Medical Department, University Hospital of Ferrara Arcispedale Sant’Anna, 44124 Ferrara, Italy

**Keywords:** obesity, MICT, HIIT, human, skeletal muscle, mitochondria, perilipins

## Abstract

Obesity is a complex condition characterized by abnormal and excessive fat accumulation, resulting in an increased risk for severe health problems. Skeletal muscles play a major role in movement and fat catabolism, but the insulin resistance that comes with obesity makes it difficult to fulfill these tasks. In this study, we analyse two types of training protocols, moderate intensity continuous training (MICT) versus high intensity interval training (HIIT), in a cohort of obese subjects to establish which muscle adaptations favour fat consumption in response to exercise. Mitochondria play a role in fat oxidation. We found protein upregulation of mitochondrial biomarkers, TOMM20 and Cox-4, in HIIT but not in MICT, without detecting any shifts in fibre composition phenotype of the vastus lateralis in both training groups. Interestingly, both MICT and HIIT protocols showed increased protein levels of perilipin PLIN2, which is involved in the delivery and consumption of fats. HIIT also augmented perilipin PLIN5. Perilipins are involved in fat storage in skeletal muscles and their upregulation, along with the analysis of circulatory lipid profiles reported in the present study, suggest important adaptations induced by the two types of training protocols that favour fat consumption and weight loss in obese subjects.

## 1. Introduction

Adipose tissue is an important site for lipid storage as well as endocrine functions. However, in obese people, the hypertrophy of adipocytes and accumulation of non-esterified fatty acids, accompanied by high levels of inflammation, can eventually lead to hypoxia and other diseases that exacerbate the condition [1]. For this reason, an effective training protocol that mobilizes triacylglycerols (TAGs) and improves fat oxidation in obese people is the most approachable intervention that can be used, alongside nutrition. In our previous study [2], we evaluated the effectiveness of high-intensity interval training (HIIT) and moderate-intensity continuous training (MICT) in boosting fat oxidation in obese subjects, and in the ability to maintain these results over time. We evaluated the ability of HIIT in establishing a high fat oxidation rate, not only after the training period at high V’O2 peaks, but also after a follow-up period, which would indicate the ability to maintain these benefits over time [2].

Skeletal muscle fibres play a role in fatty acid consumption through β-oxidation, producing energy as ATP and consuming both endogenous and exogenous lipids. Mitochondrial metabolism is aerobic and active during exercise. β-oxidation is activated alongside mitochondrial metabolism to support aerobic metabolism during physical activity [3]. Skeletal muscles require fatty acids (FAs). FAs are provided by circulating lipids released by feeding, such as TAGs in chylomicrons, lipids released by the liver as TAGs in very-low density lipoproteins (VLDL), and albumin-associated lipids mobilized by adipose tissue [4]. Exercise has been associated with increased fatty acid translocase/CD36 on the sarcolemma, acting as FA transporters and increasing TAG availability during intense workloads [5]. FAs can be immediately used during exercise or can be stored in intramyocellular structures called lipid droplets (LDs), located close to the mitochondria [6]. Type I skeletal muscle fibres are richer in LDs than type II, surely explained by their higher oxidative capacity [7]. However, this distribution appears to be affected in obesity, independent of fibre type and oxidative capacity of skeletal muscle fibres [8]. In fact, reduced FA oxidation capacity has been reported in obese people, who rely more on carbohydrate oxidation during fasting [9,10]. LDs represent a fast TAG source in skeletal muscle fibres. During exercise, they are rapidly converted into fatty acids, becoming available for mitochondrial oxidation. The coating proteins of LDs are called perilipins. They are structurally and metabolically involved in FA oxidation, especially in forming contacts between LDs and mitochondria in skeletal muscle fibres, which increases the use of endogenous FAs as a source of energy during exercise [11]. The capacity of skeletal muscle to burn lipids better, leading to higher oxidative mitochondrial metabolism and improved lipid handling at the circulatory level, would be greatly beneficial for obese people.

Thus, in the present study, we aimed to better characterize mitochondrial biomarkers and local, as well as systemic, fat management that contributes to a high fat oxidation rate in HIIT-trained obese people, in comparison with MICT. In our previous study, we found that HIIT training increased FAs oxidation rate at high V’O2 peaks, showing that the major contributor was increased mitochondrial respiration and functioning [2]. Here, we evaluated mitochondrial mass biomarkers: translocase of the outer mitochondrial membrane 20 (TOMM20) and cytochrome c oxidase subunit-4 (Cox-4). TOMM20 is an important component of the mitochondrial protein import machinery, not only for the intermembrane space, but also for matrix import in the skeletal muscle [12]. Generally, TOMM20 is recognized as a good mitochondrial mass biomarker that increases with the mitochondrial pool, due to mitochondrial biogenesis and mitochondrial enlargement. Cox-4 is a nuclear-encoded subunit of cytochrome c oxidase that may play a role in regulating its ability to create a proton gradient across the inner mitochondrial membrane. Evaluating Cox-4 protein levels is a good biomarker of mitochondrial efficiency and functionality, especially for oxidative skeletal muscle fibres involved in FA consumption.

## 2. Results

The subjects recruited in the study did not show different vital parameters, as shown in Table 1 and in the parallel article by D’Amuri et al., 2021 [13].

The intervention had an effect on both groups, with both reporting weight loss, as shown in Table 2 and in D’Amuri et al., 2021 [13].

To better comprehend the origin of the weight loss and whether there was a difference in molecular mechanisms between the training protocols, we analysed protein levels of mitochondrial and lipid droplet biomarkers, along with an evaluation of skeletal muscle fibre composition, to highlight possible shifts in fibre type composition by the end of the training period.

### 2.1. Evaluation of Mitochondrial Biomarkers

**Figure 1:**
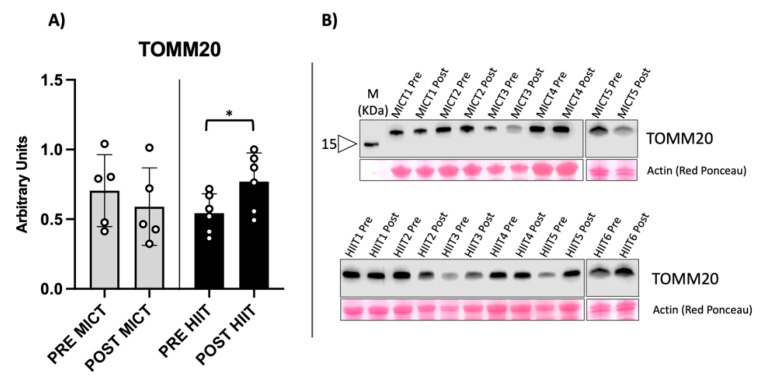
Evaluation of TOMM20 protein content as mitochondrial mass biomarker. (**A**) The graph shows a statistically significant increase in HIIT and no significantly variation in MICT. (**B**) Representative immunoblot obtained from the vastus lateralis of an obese subject. Western blot results are derived from two separate blots as shown in the figure. * = *p*-value < 0.05 (*p* = 0.049 in HIIT).

**Figure 2:**
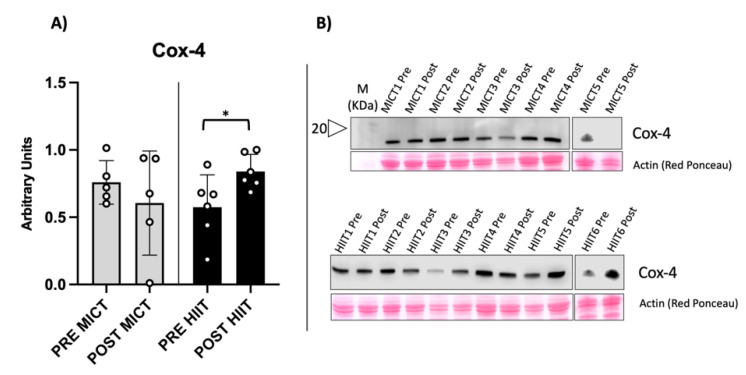
Evaluation of Cox-4, component of electron transport chain, as mitochondrial functionality biomarker. (**A**) The graph shows a statistically significant increase in HIIT and unchanged variation in MICT. (**B**) Representative immunoblot obtained from the vastus lateralis of an obese subject. Western blot results are derived from two separate blots as shown in the figure. * = *p*-value < 0.05 (*p* = 0.039 in HIIT).

We found significantly increased levels of both TOMM20 and Cox-4 in HIIT-trained obese people, compared with the unchanged levels found in MICT-trained counterparts (Figure 1 and Figure 2).

### 2.2. Myosin Heavy Chain Isoform Characterization

**Figure 3:**
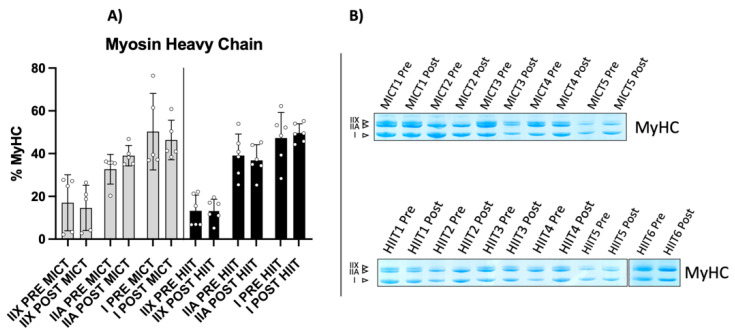
Myosin heavy chain isoform distribution. (**A**) Histogram showing myosin heavy chain isoform distribution for each human isoform, in MICT and HIIT, respectively. (**B**) Representative 8% acrylamide/bis-acrylamide gel obtained from both training groups. MyHC results are derived from two separate gels as shown in the figure.

HIIT-trained obese people showed an augmented mitochondrial pool. Mitochondria were enriched in oxidative muscle fibres (type I); thus, we evaluated whether a fibre-type shift occurred in obese subjects. As shown in Figure 3, there were no statistically significant changes in either training group. However, a marked but not significant trend of increased type IIA muscle fibres was present in MICT-trained obese subjects, with a huge Cohen’s effect size of 1.3. Conversely, in the HIIT-trained population, we observed an increase in the oxidative myosin heavy chain (type I), and although it was not significant, it had a medium effect size of 0.5. Furthermore, there was a nonsignificant trend of decreased type I muscle fibres in MICT and type IIA muscle fibres in HIIT, both with the same small effect size (d = 0.3). These data may indicate the early stages of a muscle fibre-type shift toward type IIA in MICT and type I in HIIT, which would be consistent with our findings for mitochondrial biomarkers, and also with findings in the literature, where obese mice showed a pronounced shift toward fast fibre types compared with controls [14].

### 2.3. Evaluation of Muscular Lipid Droplet Biomarkers

**Figure 4:**
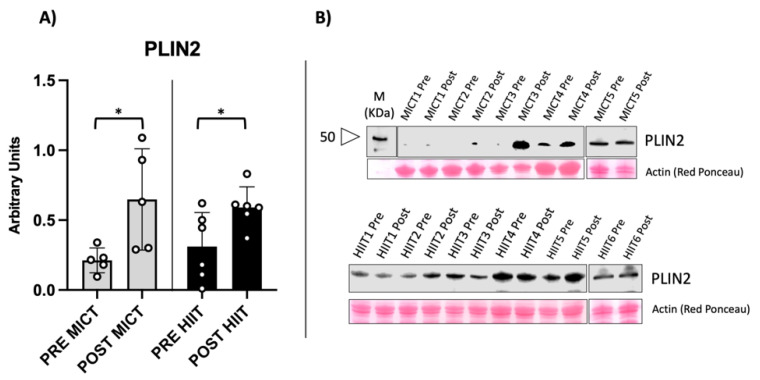
Perilipin 2 protein content. PLIN2 is a protein involved in LD formation. (**A**) Histogram showing significant increase in PLIN2 expression in both groups. (**B**) Example of the immunoblot obtained from vastus lateralis. Western blot results are derived from two separate blots as shown in the figure. * = *p*-value < 0.05 (*p* = 0.031 in MICT and 0.037 in HIIT).

The increased mitochondrial mass raised the question of whether increases in LD structures and lipid availability were among the factors that favoured fat mass loss. We evaluated two perilipin isoforms: perilipin isoform 2 (PLIN2) and perilipin isoform 5 (PLIN5), which are involved in the biogenesis of lipid droplets and in the association of droplets with mitochondria, respectively [14,15]. As shown in Figure 4, PLIN2 protein content significantly increased in both training protocols, suggesting an eventual increase in LD structures in skeletal muscle fibres.

**Figure 5:**
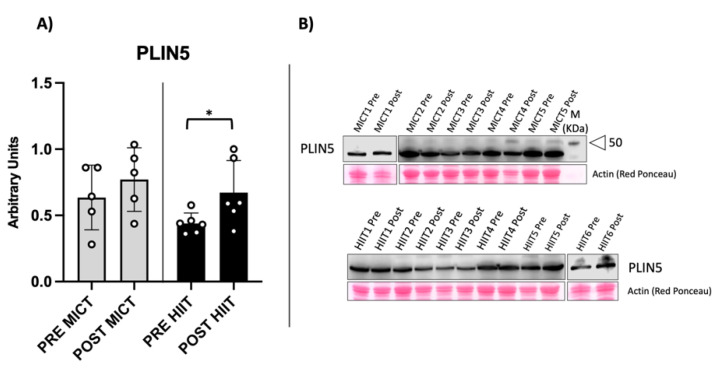
Perilipin 5 protein content. PLIN5 is a protein involved in LDs formation, as well as in establishing contacts with mitochondria. (**A**) Histogram showing significant increase in PLIN5 expression in HIIT group. (**B**) Example of the immunoblot and marker of molecular weight. Western blot results are derived from two separate blots as shown in the figure. * = *p*-value < 0.05 (*p* = 0.049 in HIIT).

PLIN5 protein expression increased and reached significance in the HIIT group but not the MICT group, as shown in Figure 5. Thus, both training protocols favoured an increase in perilipin content, which in turn can promote contact between lipid droplets and mitochondria, sustaining fatty acid oxidation during exercise.

### 2.4. Evaluation of Circulating Lipid Profile

**Figure 6:**
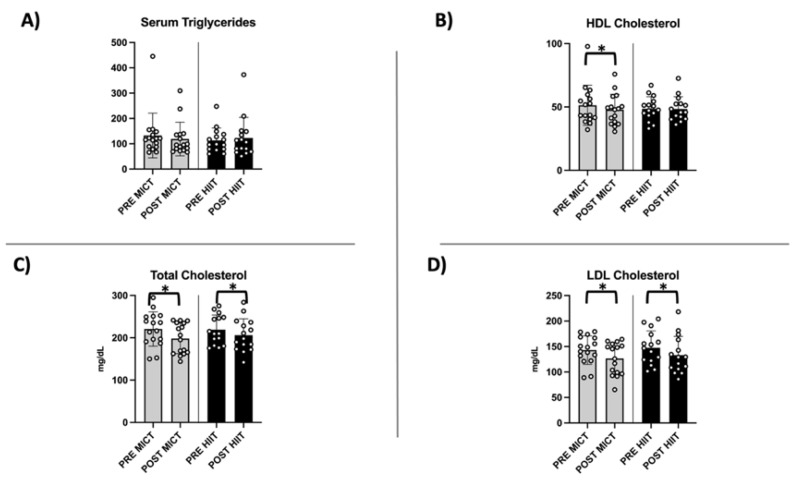
Circulating lipid profile. The data are shown as the average ± standard deviation. Comparison between MICT and HIIT groups in levels of (**A**) serum triglycerides, (**B**) HDL cholesterol (*p*-value 0.045 for MICT), (**C**) total cholesterol (*p*-value 0.01 and 0.03, respectively for MICT and HIIT), and (**D**) LDL cholesterol (*p*-value 0.02 and 0.01, respectively for MICT and HIIT) is shown. * = *p*-value < 0.05.

The evaluation of the circulating lipid profiles of both groups showed an improvement in the medical parameters used for to prevent cardiovascular risk, in accordance with our previous study [13]. In fact, both training protocols reduced LDL and total cholesterol (Figure 6C,D). The effect on triglycerides was less effective, and did not reach significance, consistent with the animal model [16].

## 3. Discussion

In our previous studies, we showed that a 3-month HIIT program was effective in promoting peak V′O2 during exercise and increasing the fat oxidation rate during submaximal exercise, maintaining these results until after the follow-up period [2,17]. Both HIIT and MICT were found to be less effective in decreasing serum triglyceride levels, with differences not reaching significance, which was consistent with findings in an animal model [16]. However, in comparison with the more common MICT training protocol, we suggest that a HIIT training protocol could be the most effective in ameliorating cardiovascular risk factors and physical capacities [2,17,18,19]. Even though both training protocols resulted in similar weight loss [2,17], we found that they induced different adaptations at a molecular level. In the current study, we found increased protein expression of TOMM20, Cox-4, and PLIN5 in the HIIT group only, and increased expression of PLIN2 in both training protocols.

PGC-1α (peroxisome proliferator-activated receptor gamma coactivator 1-alpha) is the master regulator of mitochondrial biogenesis, activated during exercise and results in the expression of markers, such as TOMM20 and Cox-4, which are related to mitochondrial mass and function, respectively [20]. Increases in PGC-1α gene expression have been reported for both obese and healthy populations after undergoing HIIT training; similar findings have been found in animal models [21,22,23]. Consistent with these findings, our subjects showed a significant increase in TOMM20 and Cox-4 protein content following the HIIT protocol. This was notable because Cox-4 expression stimulated by leptin seems to be abrogated in obesity, as shown in an in vitro study of human myotubes [24]. The increase in these mitochondrial markers suggest increased mitochondrial biogenesis [2,25]. PGC-1α controls the metabolism of oxidative muscle fibres and drives switching processes in fibre type composition [26]. Thus, we wondered if HIIT training would make a difference in the fibre-type percentage of VL. We were unable to identify any statistically significant changes in fibre type composition, even though HIIT showed prominent switching toward slow MyHCs (type I), with a considerable Cohen’s effect size. On the other hand, MICT showed a trend of switching toward type IIA MyHCs, with a significant Cohen’s effect size, partially explaining the different mitochondrial content stimulated by each training group. Overall, HIIT appeared to induce an activation of mitochondrial biogenesis and respiration, accompanied by an early, but non-significant, trend in switching fibre-type content to the slow fibre type. On the other hand, the MICT protocol seemed to be less effective in activating mitochondrial metabolism, reflected in the trend to switch toward type IIA MyHCs, in agreement with previous studies [2,17].

Mitochondria play a role in the oxidation of FAs, an important metabolic pathway that should be activated and maintained in obesity. The demand for FAs during exercise is met by circulating plasma lipids originating from adipose tissue, as well as in the intestine from chylomicrons and in the liver from very-low-density lipoproteins (VLDL). Thus, inducing the mobilization of fatty acids from adipose tissue to skeletal muscle is crucial in obese people to favour adipose tissue catabolism. Lipid droplets are main storage sites and represent an interface for lipid oxidation during exercise, preventing any detrimental accumulation in other subcellular locations [27]. Thus, an increase in LD structures during exercise may represent an important adaptation for obese people, as shown in other pathological contexts [28,29,30,31]. Perilipins are the main structural proteins coating LDs, with an active role in metabolism. In fact, they have been classified into isoforms PLIN2, 3, 4, and 5. However, the main isoforms expressed in skeletal muscle with a regulatory role are PLIN2 and PLIN5 [21,32,33]. PLIN2 is more involved in LD formation, whereas PLIN5 has been shown to regulate the interface with mitochondria where FAs are metabolized [20,32]. We measured levels of both PLIN2 and PLIN5 and found a significant increase in both perilipins in the HIIT group, whereas only PLIN2 levels increased in the MICT group. The interpretation of these results is corroborated by the mitochondrial parameters found in the present study. Notably, mitochondrial biogenesis has also been associated with induction of PLIN2 and PLIN5 [34]. The expected increase in the mitochondrial pool of HIIT subjects may be accompanied with an increase in contact with LDs through PLIN5 [17]. On the other hand, even though MICT did not seem to induce an increase in mitochondrial biomarkers, it increased PLIN2 content and FA availability. Thus, the two different training protocols seem to act differently at a molecular level and in their promotion of FA consumption. This was also confirmed after the follow-up period where HIIT was able to maintain DNA methylation memory, preserving the results over an extended period [2]. As both MICT and HIIT were effective in promoting weight loss with different molecular mechanisms, this study may influence general clinical practice in adopting the appropriate training protocols for weight loss.

## 4. Methods

### 4.1. Subjects and Recruitment

A total of 32 obese volunteers (both men and women; for details, see Table 1) were recruited from the Exercise Physiology Laboratory of the University of Udine, where they were evaluated for medical and dietetic criteria. They were between 18 and 50 years of age and had body mass indexes (BMI) ≥ 30 kg m^−2^. They were split into two groups that received different training protocols: MICT versus HIIT. For women participants, notes about their last menstruation dates were made, and the menstrual status of all women were regular. One female from the MICT group and one from the HIIT group were menopausal. No sex differences were found [2]. No medications were used by subjects included in the study. The study was approved by the Ethics Committee of the Friuli-Venezia-Giulia Region (protocol number 1764). For details about the procedures, physical activity, and ethics, see methods in Vaccari et al., 2020 and D’Amuri et al., 2021 [2,13]. The in vivo experiments described below were performed on 5 subjects recruited for MICT and 6 subjects recruited for HIIT, due to limitations in samples. Analysis of circulating lipid profiles were performed on 16 subjects from the MICT group and 15 subjects from the HIIT group (due to missing data from one male participant from the HIIT group).

### 4.2. Skeletal Muscle Biopsies and Blood Sampling

Skeletal muscle biopsies were taken from the vastus lateralis (VL) muscle of the subjects, at baseline and after 3 months of the exercise protocol. After anaesthesia of the skin using lidocaine (2%), a small incision was made to penetrate skin and fascia, and then a biopsy sample was harvested with a microneedle (Tru-cut Histocore, 12 G, Biomed In153 instrument and product GmbH, Germany). A fragment of the sample was quickly immerged into cryopreserving solution (BIOPS) and stored at −80 °C for further analysis.

### 4.3. Determination of Circulating Lipid Profile

The circulating lipid profile was determined for all the subjects involved in the study, 16 subjects recruited for MICT, and 15 subjects recruited for HIIT. Blood samples were taken at the same time, after fasting overnight, and centrifuged in absence or presence of EDTA, to obtain serum and plasma. High density lipoprotein cholesterol (HDL-C) was measured after precipitation of apoprotein B (apo B), containing lipoproteins, whereas total cholesterol (Tot-C) and triglyceride levels were assayed in serum using the Trinder method [35]. The coefficient of variation was <2% for Tot-C and HDL-C and <5% for triglycerides for intra- and inter-batch, respectively. Low density lipoprotein cholesterol (LDL-C) plasma levels were calculated by the Friedewald formula.

### 4.4. Western Blot Analysis

Human skeletal muscle biopsies were stored in cryopreserving solution (BIOPS) at −80 °C until treatment. Muscle biopsies were homogenized and solubilized in Laemmli solution with pestle (62.5 mM Tris-HCl pH 6.8, 2.3% SDS, 10% glycerol), in the presence of protease inhibitors at the suggested concentration (Protease Inhibitor Cocktail 100X #5871) [36], and heated at 65 °C for 3 min to allow for the complete resuspension of proteins. Protein concentration was determined by the Folin-Lowry method, using BSA as a standard [37]. The complete separation of the protein profile was obtained on home-made SDS-PAGE (4% acrylamide for stacking and 12% acrylamide for separating). In particular, 40 μg of proteins were mixed with Laemmli solution at 5% β-mercaptoethanol, boiled for 3 min at 90 °C and separated on the mini-gel system Mini-PROTEAN^®^ Tetra Handcast System (Bio-Rad). Then, proteins were transferred to a nitrocellulose membrane on the Mini Trans-Blot^®^ System (Bio-Rad) at 100 V constant for 90 min in a cold transfer buffer containing 25 mM Tris, glycine 192 mM, and 20% methanol. The membrane was blocked with 5% BSA fraction V (A6588,0100Appli Chem) in TBSt with a pH of 7.6 (Tris 0.02 M, NaCl 0.137 M and 0.1% Tween-20) at orbital shaking. Then, the membrane was incubated for 1 h at RT with primary antibodies in a blocking buffer at a specific dilution. Subsequently, the membrane was rinsed with the washing buffer and incubated with HRP-conjugated secondary antibodies in 3% Milk (A0830,0500 Appli Chem) TBSt for 2 h. Prior to detection, the membrane was washed again, and the protein signal was visualized using an enhanced chemiluminescence (ECL Star, EUROCLONE, EMP001005) method using a digital imaging system (C-DiGit^®^ Blot Scanner, LI-COR; Image Studio Lite Software analysis system). Signal normalization was performed on actin bands obtained from Red Ponceau staining on the membrane. The molecular weight of protein bands were detected with Precision Protein Strep Tactin-HRP Conjugate (161-0380 Bio-Rad) at the appropriate dilution with Precision Plus Protein WesternC Standards (161-0376 Bio-Rad), co-incubated with a secondary antibody. Details of antibodies adopted in the study are shown in Table 3.

### 4.5. MyHC Isoforms Analysis

The myosin heavy chain (MyHC) isoforms in the whole muscle protein extraction mixture (see ‘western blot analysis’ section for details) were separated and quantified, as previously described in Bamman et al. 1999, for human skeletal muscle [38]. An amount of 15 μg of total protein extraction was separated on 8% SDS-PAGE with home-made mini-gels (Mini-PROTEAN^®^ Tetra Handcast System, Bio-Rad), and electrophoresis was run at 4 °C for ~40 h at 70 V constant. Then, the gel was stained with the Colloidal Coomassie blue staining method, modified from reported references, and protein bands (corresponding to MyHC-I, MyHC-IIA and MyHC-IIX) were quantified by densitometric analysis to evaluate the relative proportion in each subject [39,40].

### 4.6. Statistical Analysis

We performed statistical analysis and graphing using GraphPad Prism 9 (GraphPad Software, San Diego CA, USA) and evaluated differences of protein levels adopting two-tailed Student’s t-test. The data met the criteria of normality for Shapiro–Wilk and Kolmogorov–Smirnov tests and were obtained at least in double for each subject and training protocol. Data were represented in means ± standard deviations and statistical differences were considered significant at *p* < 0.05.

## 5. Conclusions

In the present study, we observed that in the obese adult population, HIIT increased the protein content of mitochondrial biomarkers, TOMM20 and Cox-4. Both HIIT and MICT exercises did not induce any significant fibre-type switch. On the other hand, both HIIT and MICT protocols increased lipid droplet biomarkers, PLIN5 and PLIN2, in skeletal muscle. This could represent different adaptation of the oxidative capacity of muscle after exercise, depending on the intensity and duration.

## 6. Limitations

We recognize that the present study had some limitations. The immunoblot analysis was performed on a limited number of subjects, with five subjects recruited for MICT and six subjects recruited for HIIT. We did not perform a time course of the weight loss over the course of training; however, the aim of the study was the evaluation of final results after 12 weeks of training.

## Figures and Tables

**Table 1:** Baseline parameters of the subjects recruited for MICT and HIIT.

Variable	MICT	HIIT	*p*-Value
	*Number*	*Number*	
**Subjects**	16	16	
**Sex, Men/Women**	9/7	8/8	0.723
**Smoker, never/in the past/current**	12/4/0	9/4/1	0.553
**Hypertension medication, no/yes**	16/0	16/0	
**Hyperlipidemia medication, no/yes**	16/0	16/0	
**Diabetes medication, no/yes**	16/0	16/0	
	***Means ± SD***	***Means ± SD***	
**Age *(years)***	37 ± 9	40 ± 7	0.335
**Weight (kg)**	107.1 ± 17.4	103.4 ± 10.9	0.479
**BMI (kg/m^2^)**	36.1 ± 5.1	35.1 ± 3.6	0.514
**VO_2_peak (mL)**	3016.3 ± 782.7	2888.7 ± 706.2	0.632
**VO_2_peak/weight (mL/kg)**	28.3 ± 6.5	27.8 ± 5.6	0.824

MICT, moderate intensity continuous training. HIIT, high-intensity interval training. BMI, body mass index. VO_2_peak, oxygen uptake during peak exercise. *Notes:* Table 1 *modified from D’Amuri et al., 2021* [13].

**Table 2:** Effect of training protocol (MICT vs. HIIT) on body weight, anthropometry, and body composition.

Outcome Measures		Intervention Type	BDC	PTDC	*p*-Value Paired Samples *t*-Test	Intervention x Intervention Type Effects MD (95% CI)	*p*-Value GLM Change Between Groups
**Weight (kg)**		***MICT***	107.1 ± 17.4	101.1 ± 17.9	**0.001**	−6.0 (−9.0–−3.0)	0.860
		***HIIT***	102.8 ± 11.0	97.1 ± 10.2	**<0.001**	−5.7 (−8.3–−3.1)	
**BMI (kg/m^2^)**		***MICT***	36.1 ± 5.1	33.9 ± 4.8	**0.001**	−2.1 (−3.2–−1.1)	0.696
		***HIIT***	35.0 ± 3.7	33.1 ± 4.2	**<0.001**	−1.9 (−2.7–−1.0)	
**Waist (cm)**	***Men***	***MICT***	116.5 ± 17.1	109.6 ± 17.7	0.369	−1.8 (−6.1–2.5)	**0.039**
		***HIIT***	117.1 ± 9.5	101.2 ± 9.0	**<0.001**	−8.5 (−10.9–−6.1)	
	***Women***	***MICT***	108.6 ± 8.1	90.1 ± 11.5	**0.054**	−4.4 (−8.9–0.1)	0.774
		***HIIT***	110.6 ± 7.6	93.4 ± 10.4	**0.043**	−2.4 (−4.7–−0.1)	
**Hip (cm)**	***Men***	***MICT***	120.3 ± 10.4	118.7 ± 12.8	0.280	−1.5 (−4.6– 1.5)	0.102
		***HIIT***	116.4 ± 6.8	111.9 ± 5.7	**0.001**	−4.5 (−6.4–−2.5)	
	***Women***	***MICT***	126.7 ± 11.6	118.0 ± 9.9	**0.011**	−8.7 (−14.7–−2.8)	0.155
		***HIIT***	124.4 ± 6.4	119.9 ± 10.0	**0.020**	−4.5 (−8.1–−1.0)	
**FM (kg)**		***MICT***	37.7 ± 10.9	32.4 ± 9.1	**<0.001**	−5.3(−7.8–−2.8)	0.919
		***HIIT***	38.4 ± 8.2	32.9 ± 10.1	**0.001**	−5.5(−8.3–−2.6)	
**FM (%)**		***MICT***	35.4 ± 8.9	32.5 ± 8.3	**0.001**	−2.9(−4.4 –−1.4 )	0.619
		***HIIT***	37.3 ± 7.7	33.7 ± 9.2	**0.006**	−3.6(−5.9 –−1.2 )	
**FFM (kg)**		***MICT***	69.4 ± 15.5	65.1 ± 11.7	0.127	−5.3 (−7.8–−2.8)	0.735
		***HIIT***	68.6 ± 16.3	64.7 ± 11.0	0.641	−5.5 (−8.3–−2.6)	
**FFM (%)**		***MICT***	64.6 ± 8.9	67.4 ± 8.3	**0.002**	2.8 (1.3– 4.4)	0.554
		***HIIT***	62.3 ± 7.7	66.0 ± 9.4	**0.008**	3.7 (1.2– 6.3)	

MICT, moderate intensity continuous training. HIIT, high-intensity interval training. BDC, baseline data collection. PTDC, post training data collection. GLM, general linear model for repeated measures; BMI, body mass index. FM, fat mass. FFM, fat-free mass. *Notes:* Table 2 *modified from D’Amuri et al., 2021* [13].

**Table 3:** Antibodies adopted in the study.

Antibody	Code	Saturation	Ab Dilution
Cox-4	sc-517553	5% BSA	1:1.000
TOMM20	sc-11415	5% BSA	1:1.000
PLIN-5	LS-B5964-100	5% BSA	1:40.000
PLIN-2	NB110-40877	5% BSA	1:2.000
Anti-Mouse	ab205719	3% Milk	1:10.000
Anti-Rabbit	ab205718	3% Milk	1:10.000

## Data Availability

The data presented in this study are available in article and supplementary material.

## Supplementary Materials

The following supporting information can be downloaded at: https://www.mdpi.com/article/10.3390/metabo12090855/s1. Original Western Blot and MyHC Gel figures of Figure S1.
