# Supplementary material for: Skeletal Muscle Mitochondrial and Perilipin Content in a Cohort of Obese Subjects Undergoing Moderate and High Intensity Training
Source: Metabolites. 2022 Sep 11;12(9):855. doi: 10.3390/metabo12090855 (PMC9504635; doi:10.3390/metabo12090855)
Supplement: Supplementary file 1 [file metabolites-12-00855-s001.zip › metabolites-1903086-supplementary.pdf]

Original Western Blot and MyHC Gel figures of Figure S1.

Skeletal muscle mitochondrial and perilipins content in a cohort of obese subjects undergoing high intensity and moderate training

Giuseppe Sirago[1*], Filippo Vaccari[2,3], Stefano Lazzer[2,3], Andrea D'Amuri[4], Juana M. Sanz[5], Marco V. Narici[1], Carlo Reggiani[1], Angelina Passaro[4,6] and Luana Toniolo[1*]

TOMM20

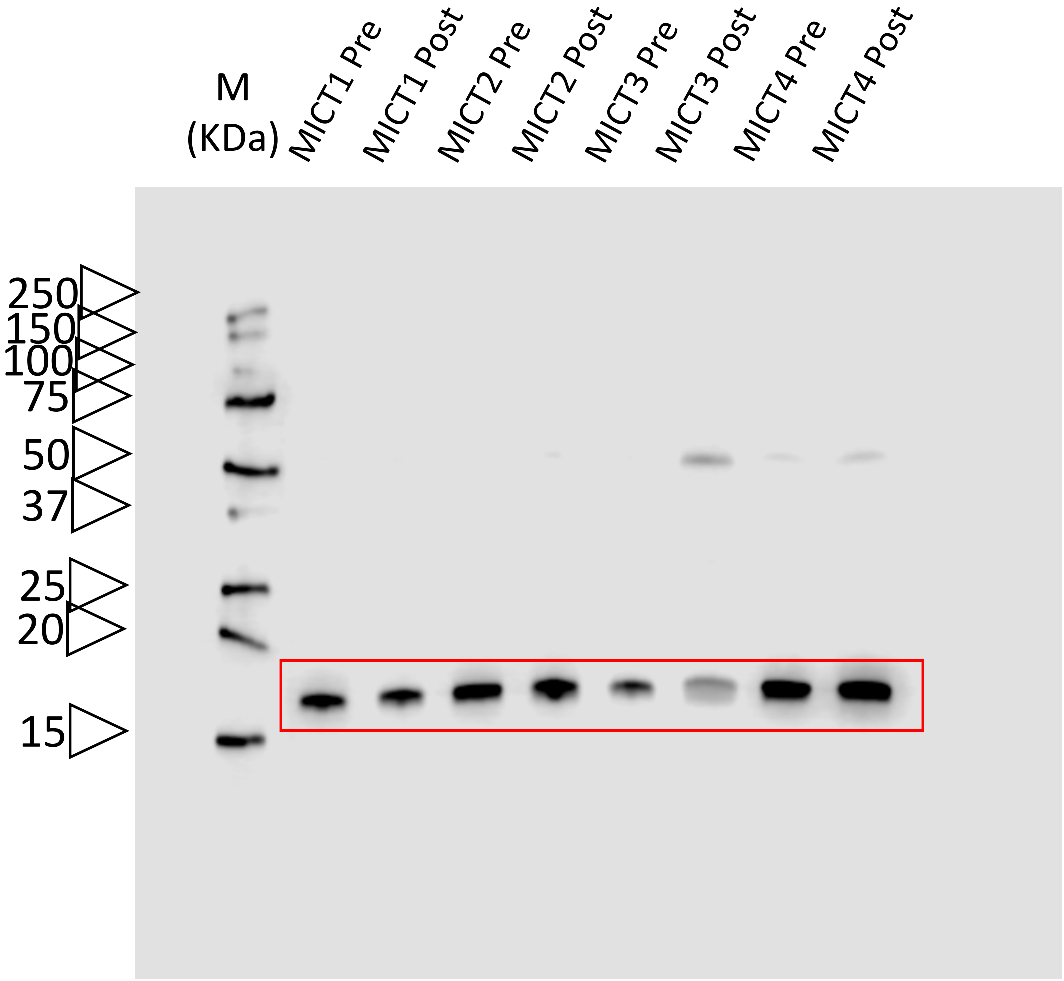

Actin in Red Ponceau

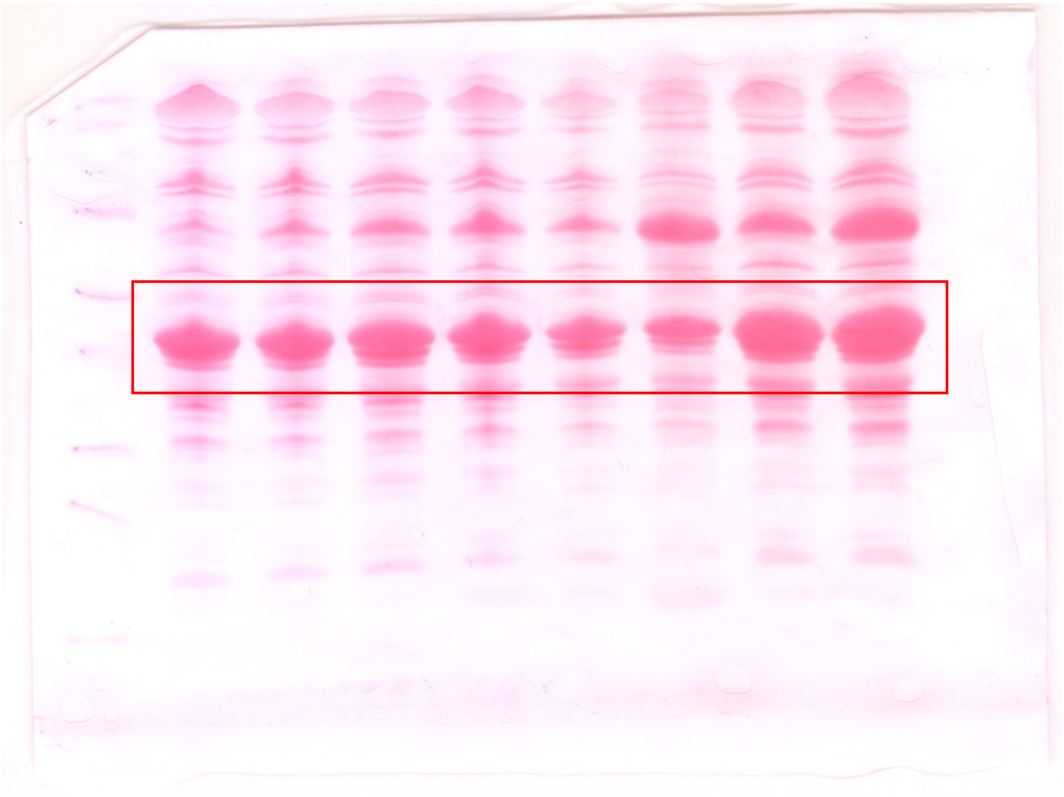

TOMM20

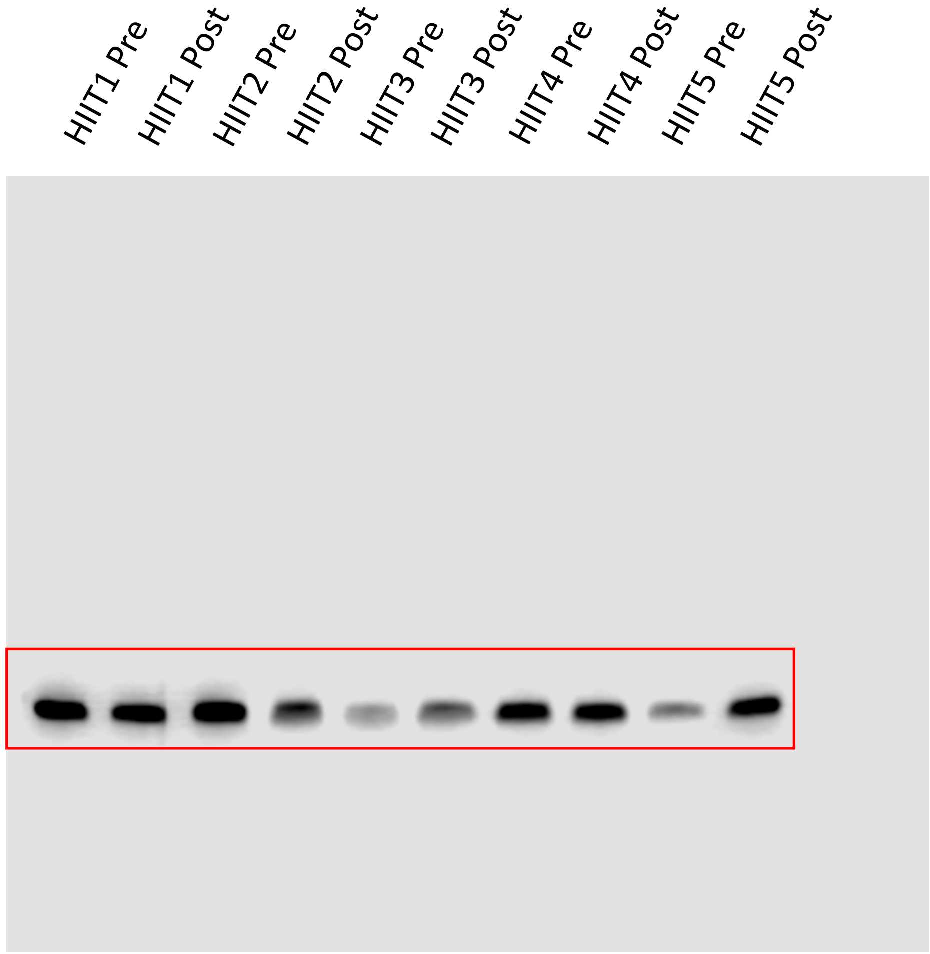

Actin in Red Ponceau

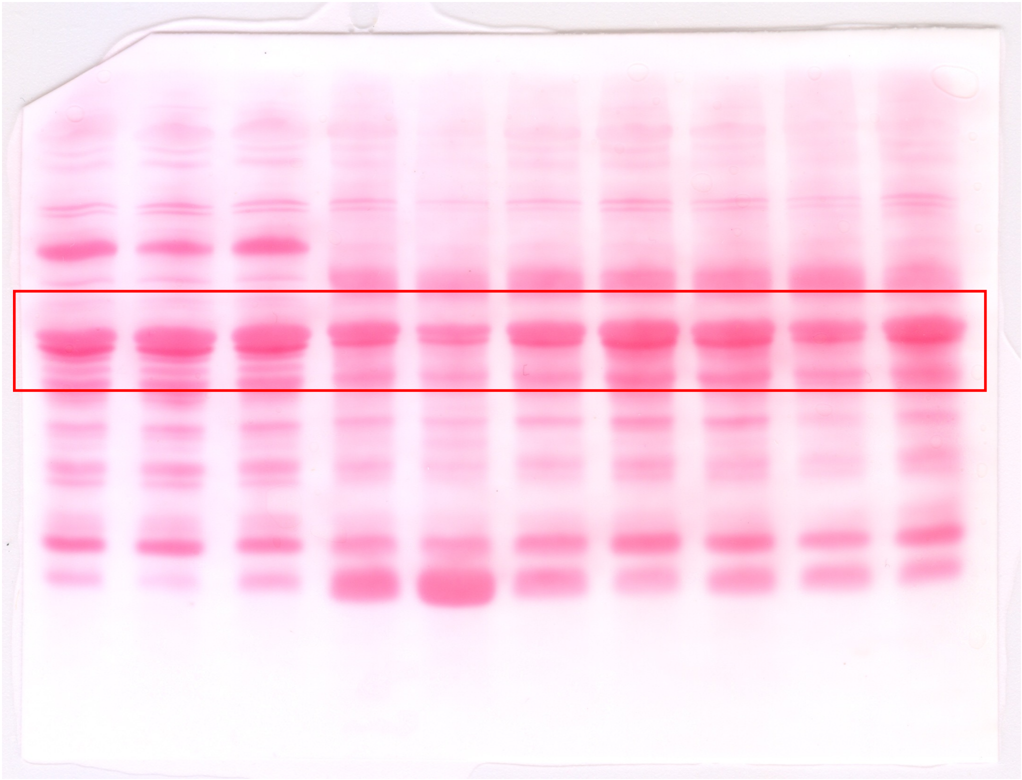

TOMM20

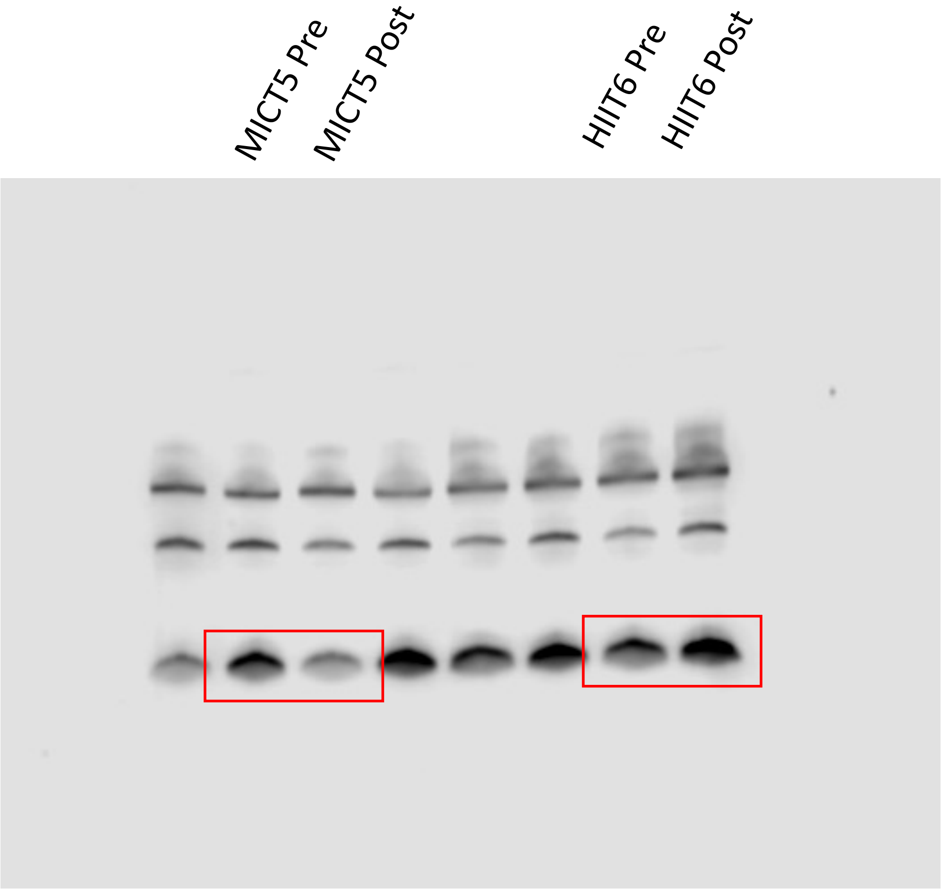

Actin in Red Ponceau

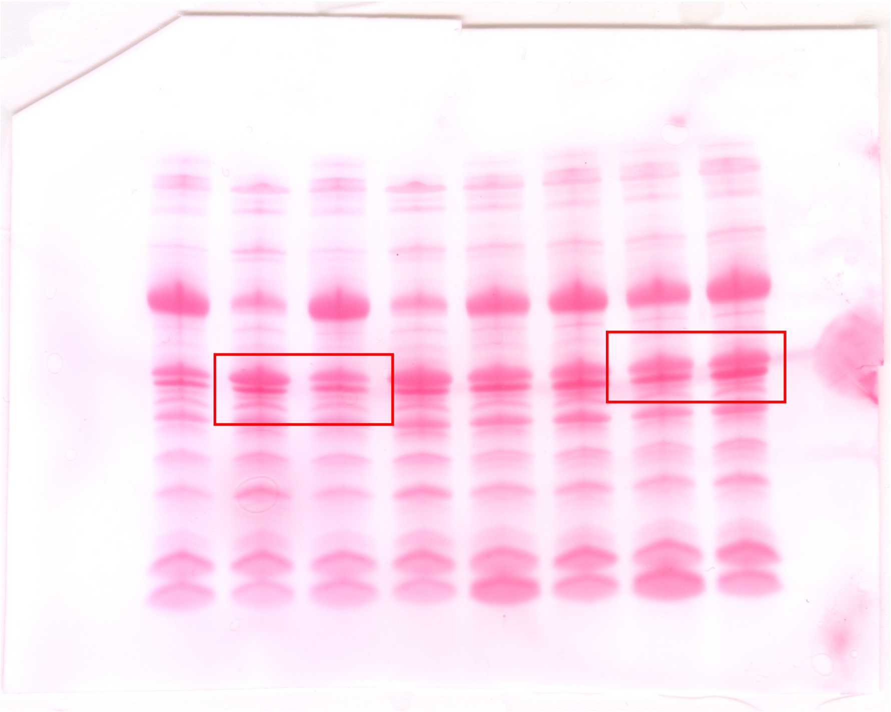

Cox4

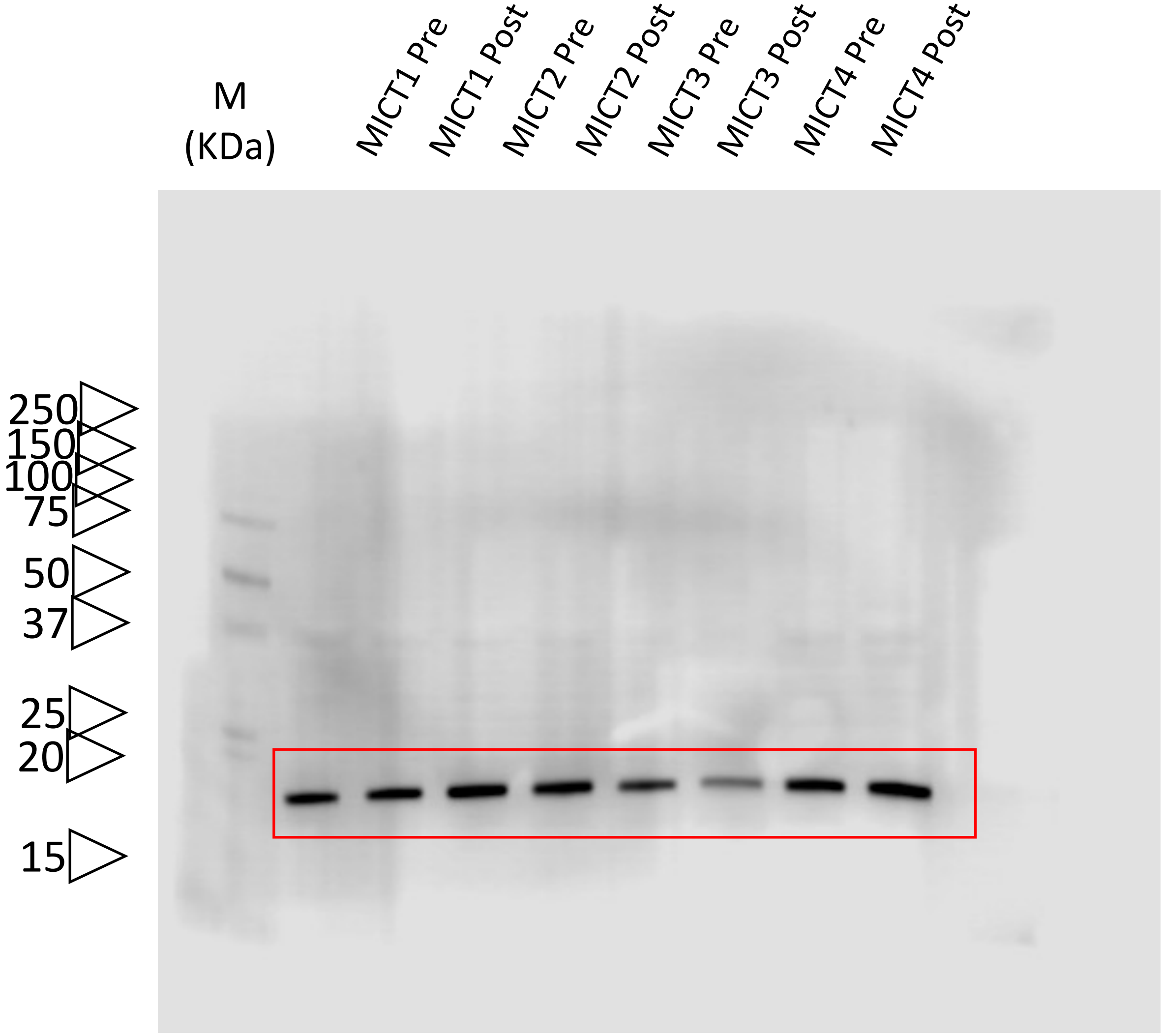

Actin in Red Ponceau

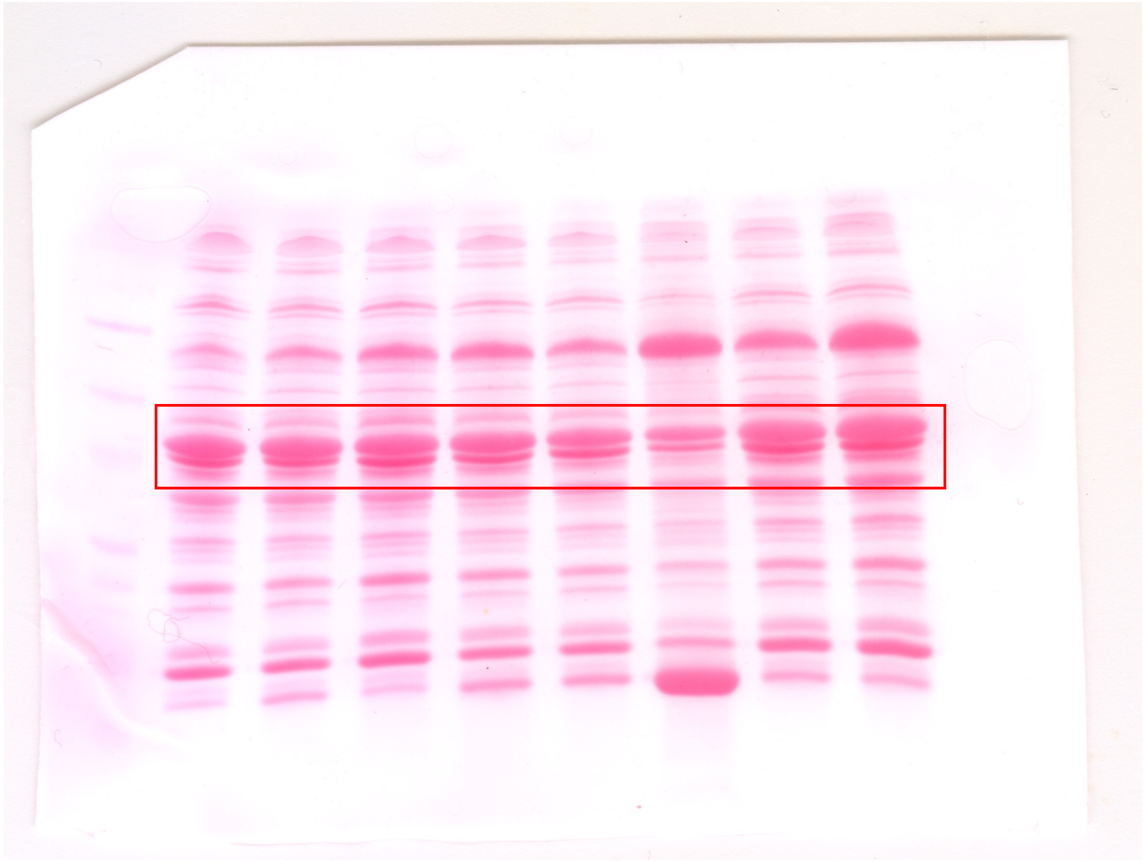

Cox4

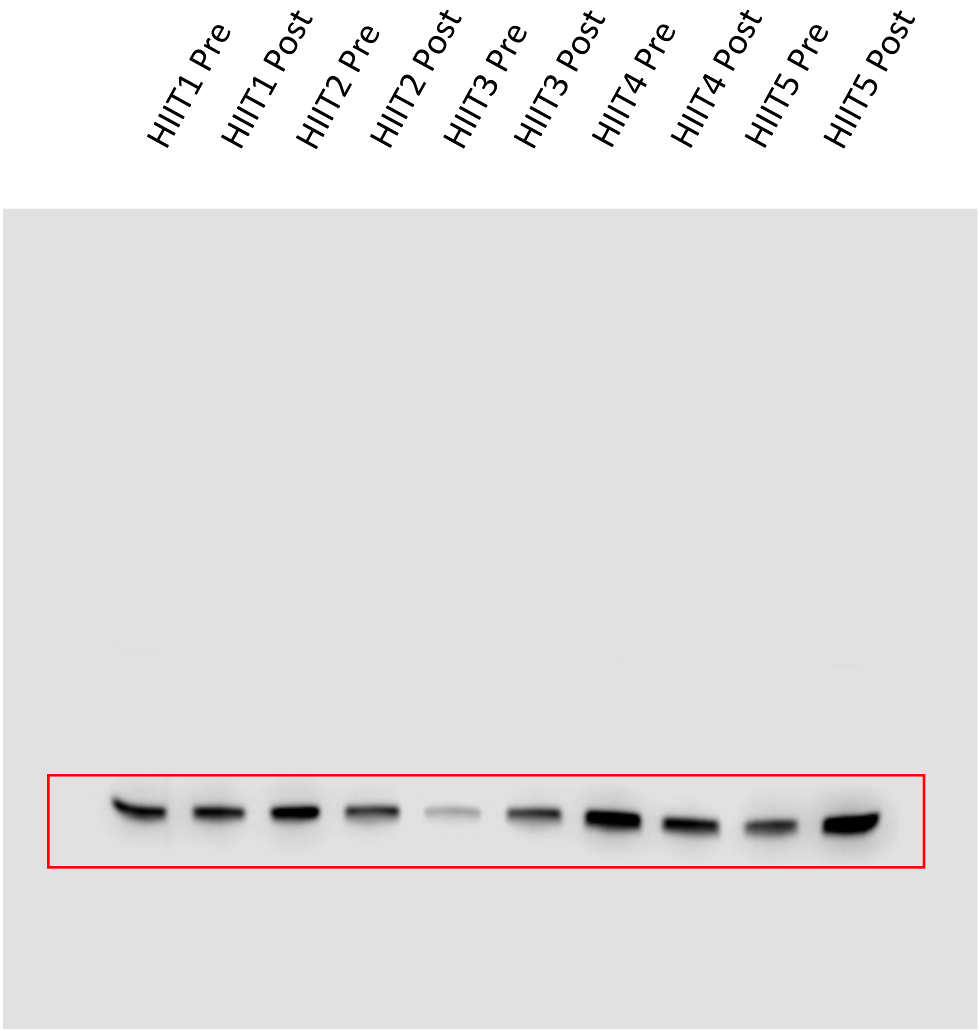

Actin in Red Ponceau

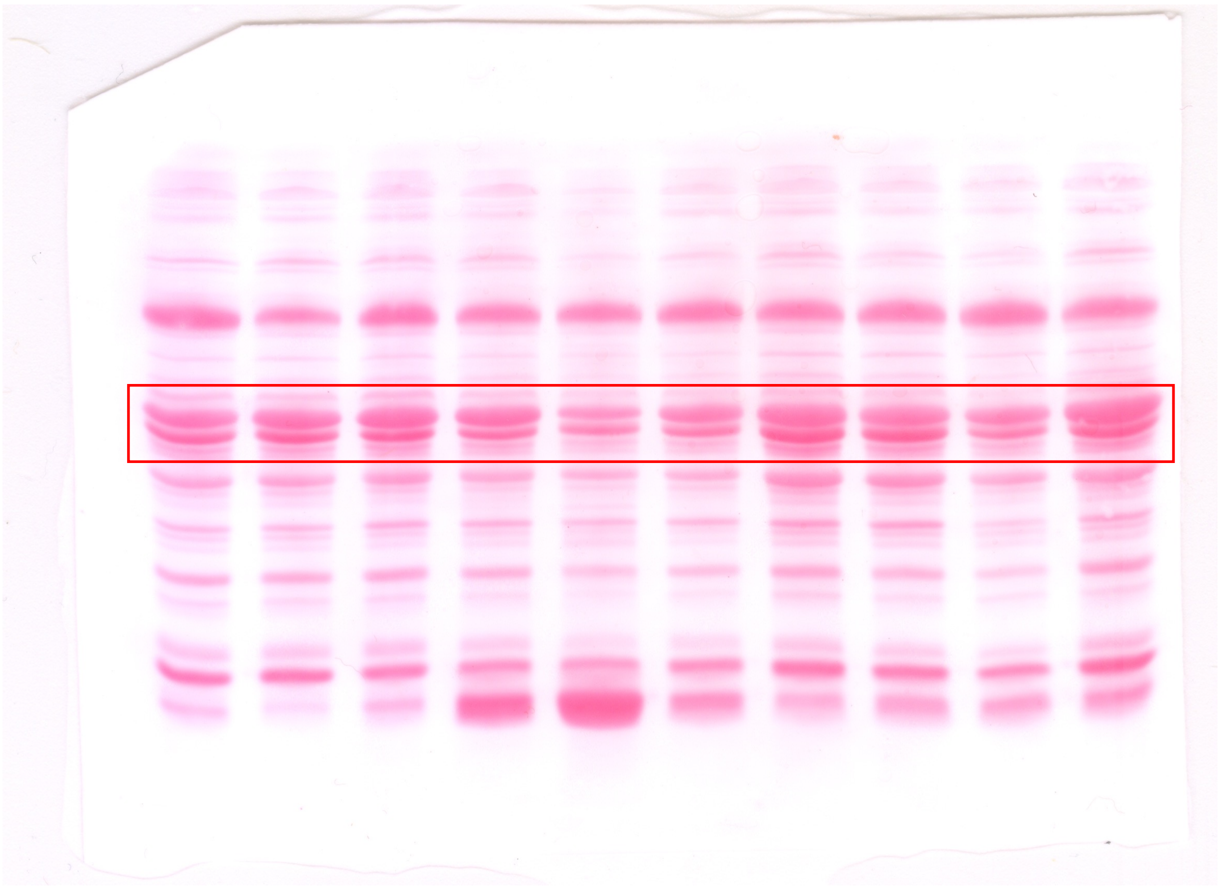

Cox4

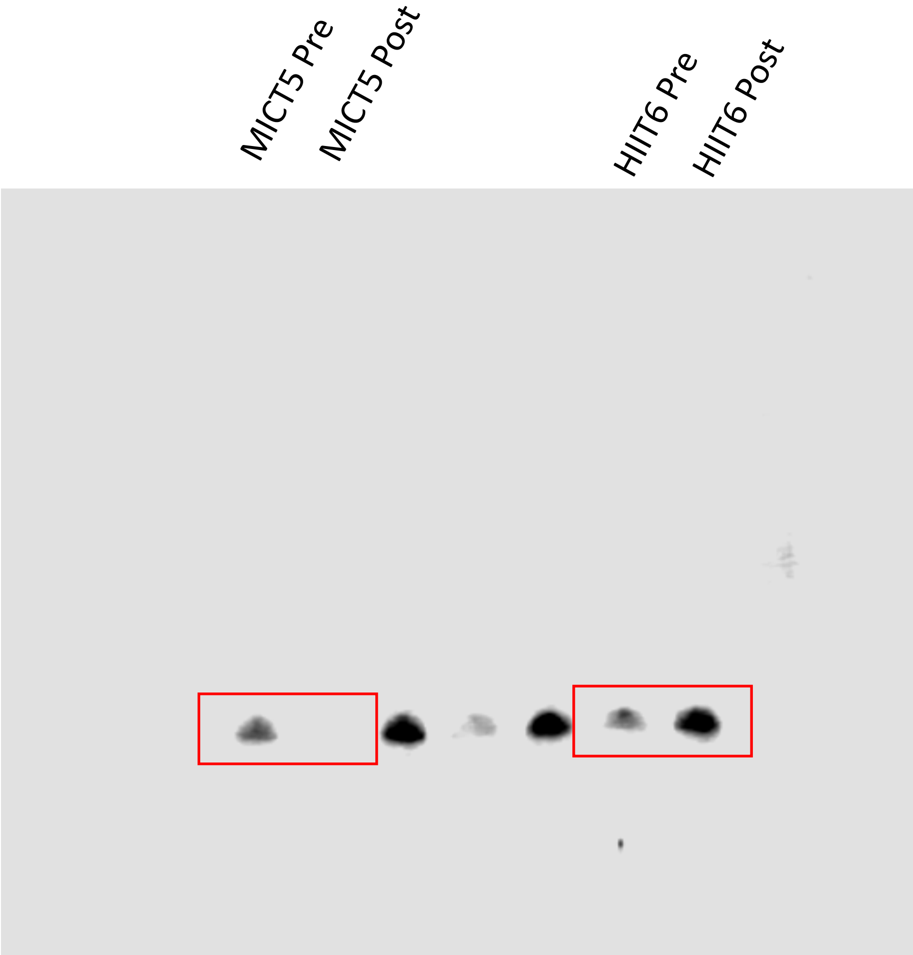

Actin in Red Ponceau

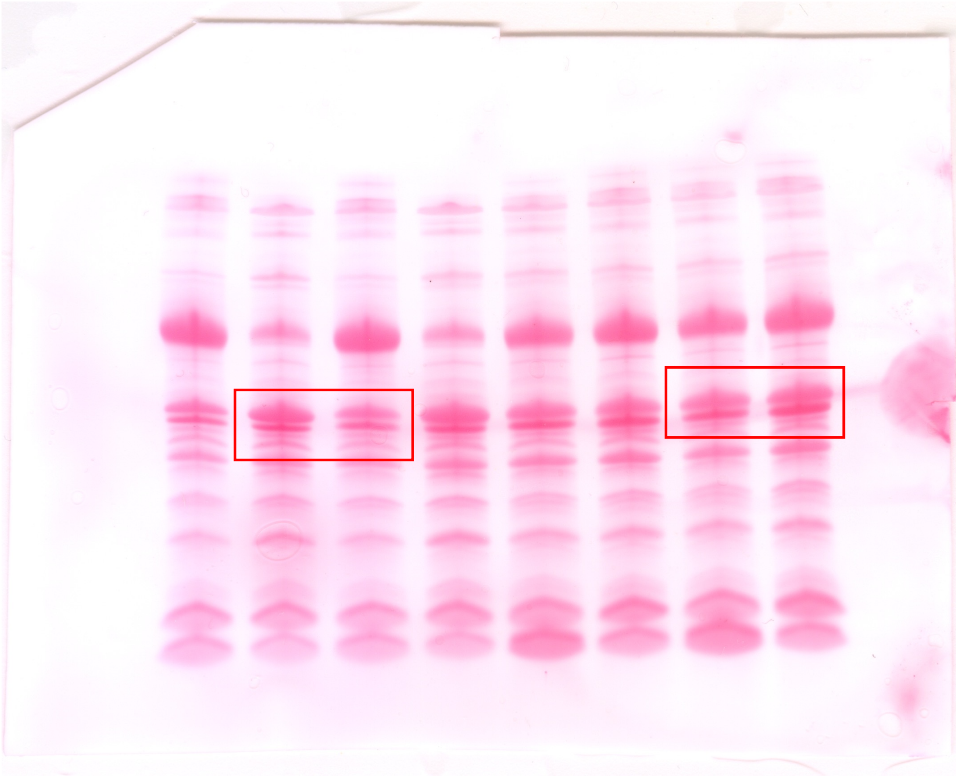

MyHC

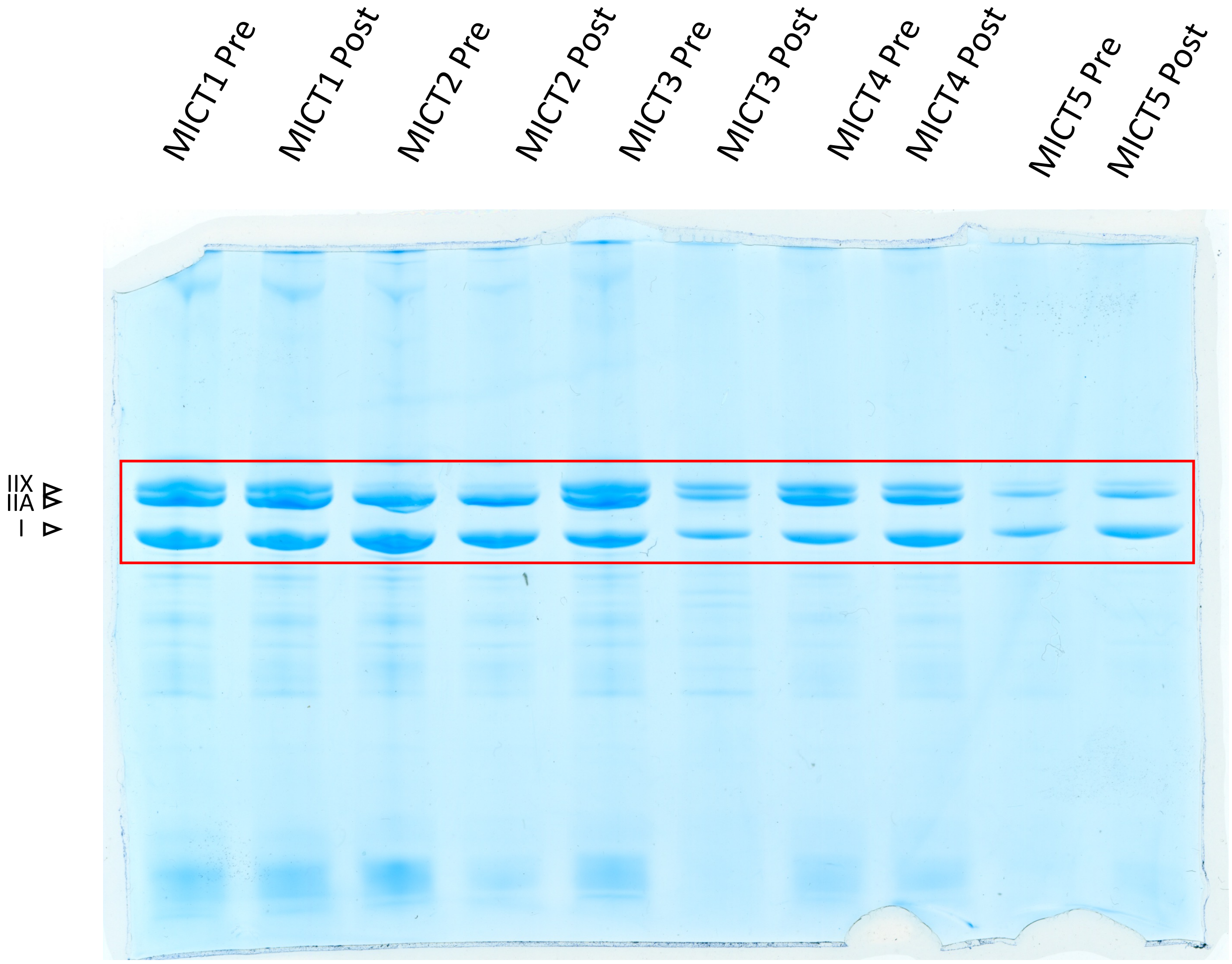
MICT1 Pre
MICT1 Post
MICT2 Pre
MICT2 Post
MICT3 Pre
MICT3 Post
MICT4 Pre
MICT4 Post
MICT5 Pre
MICT5 Post
IIX
IIA
I

MyHC

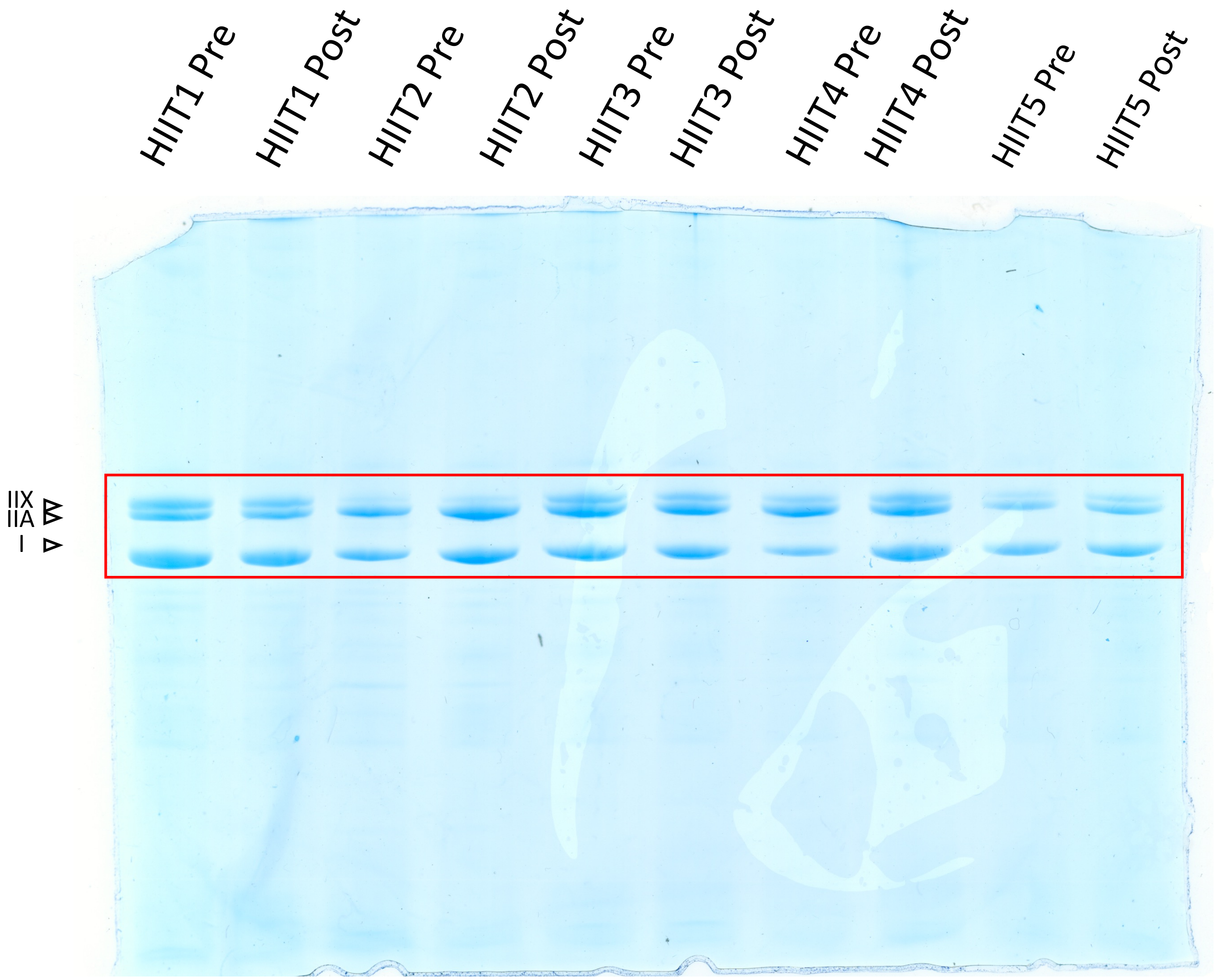
HIIT1 Pre
HIIT1 Post
HIIT2 Pre
HIIT2 Post
HIIT3 Pre
HIIT3 Post
HIIT4 Pre
HIIT4 Post
HIIT5 Pre
HIIT5 Post
IIX
IIA
I

MyHC

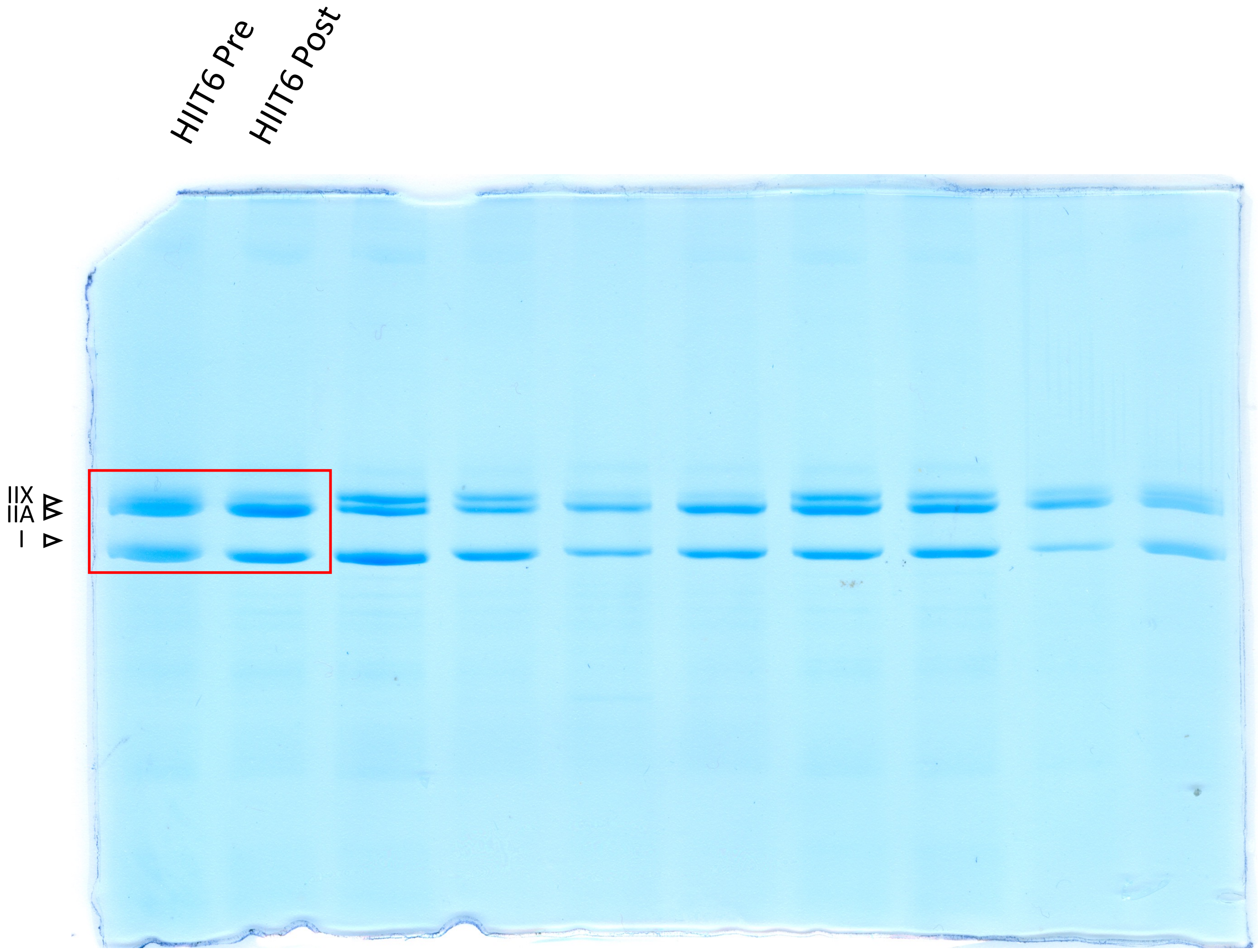

PLIN2

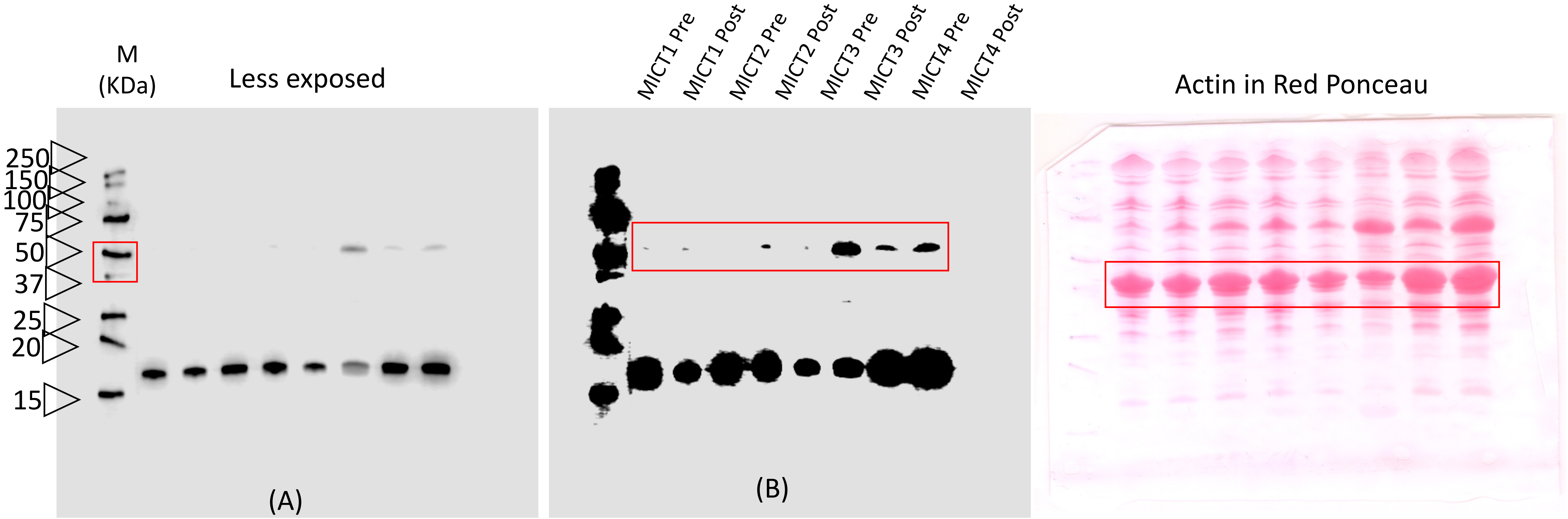

(A) and (B) are the same figure. (A) is less exposed than (B) to get a good visibility of the marker.

PLIN2

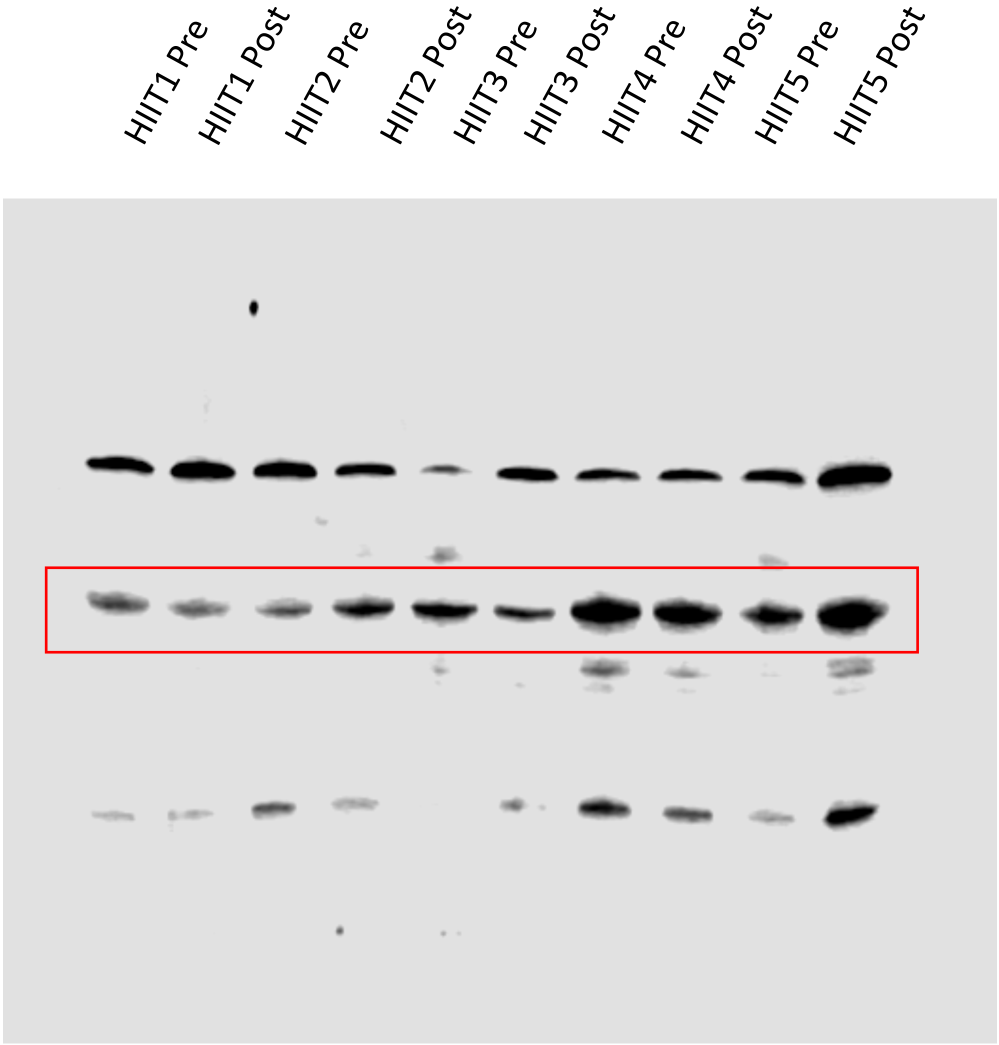

Actin in Red Ponceau

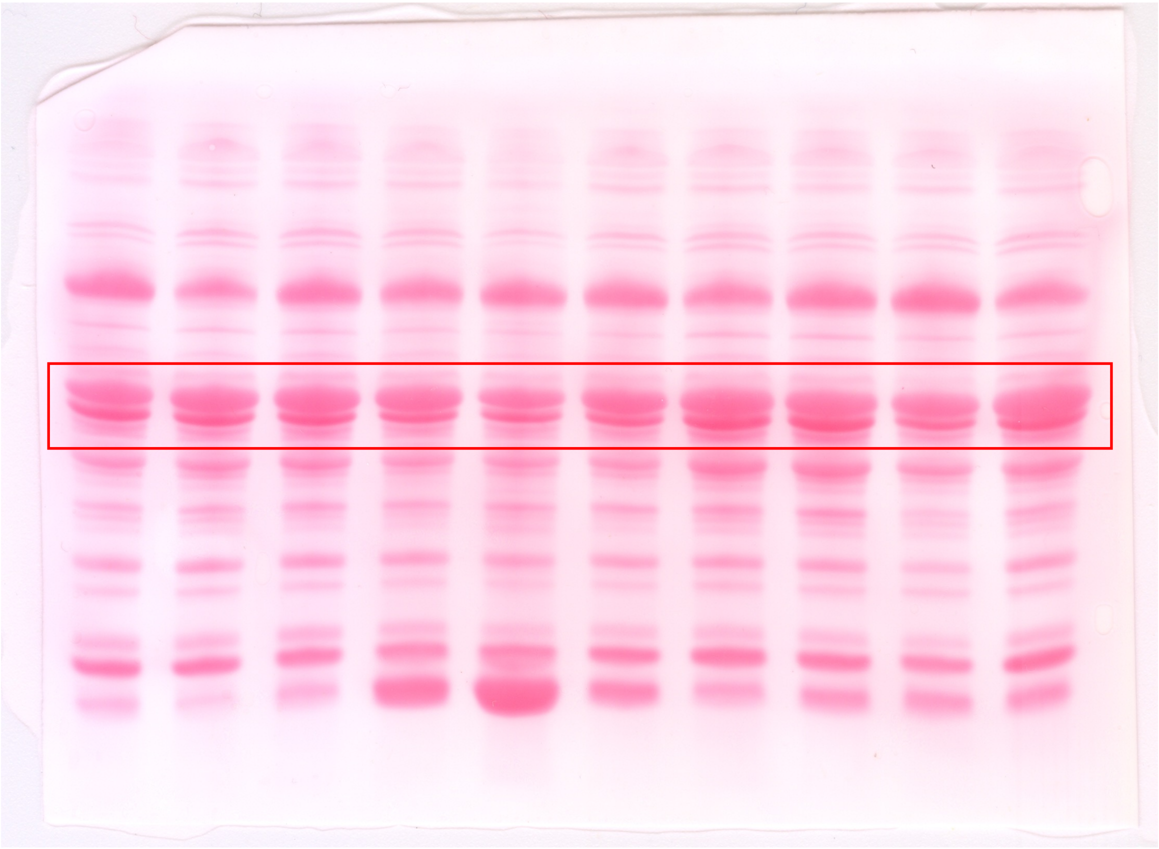

PLIN2

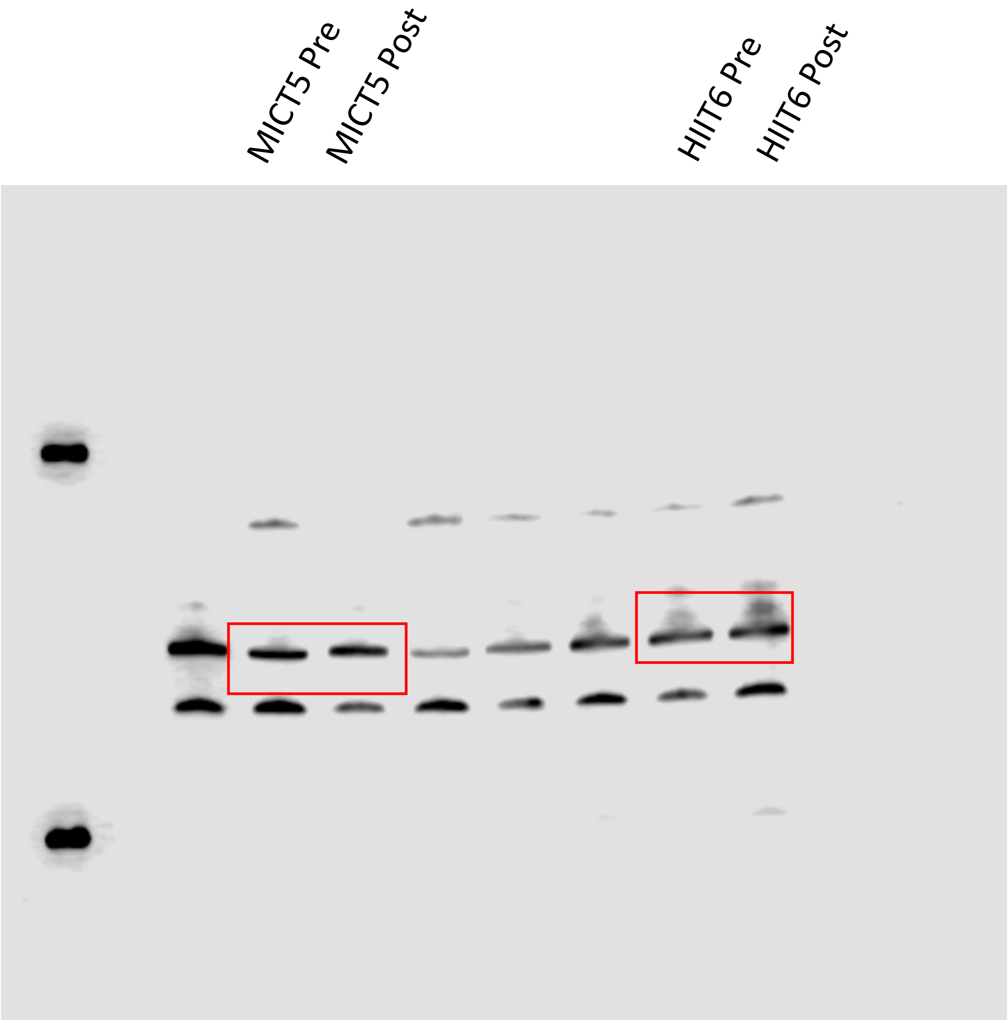

Actin in Red Ponceau

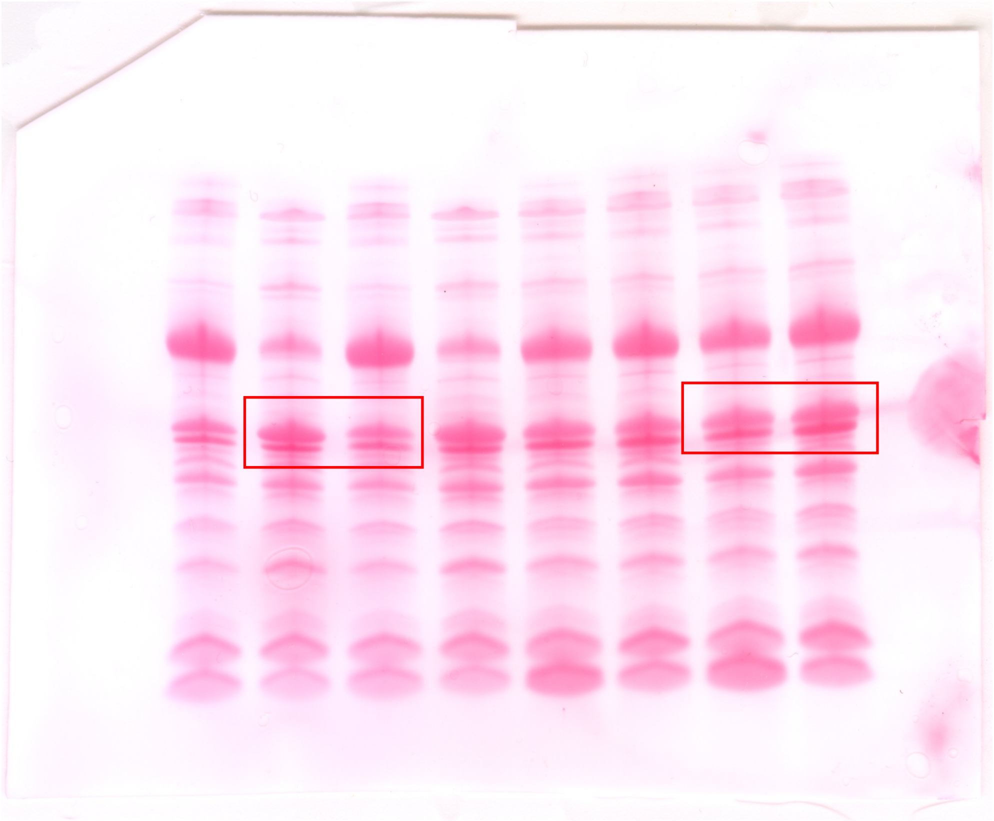

PLIN5

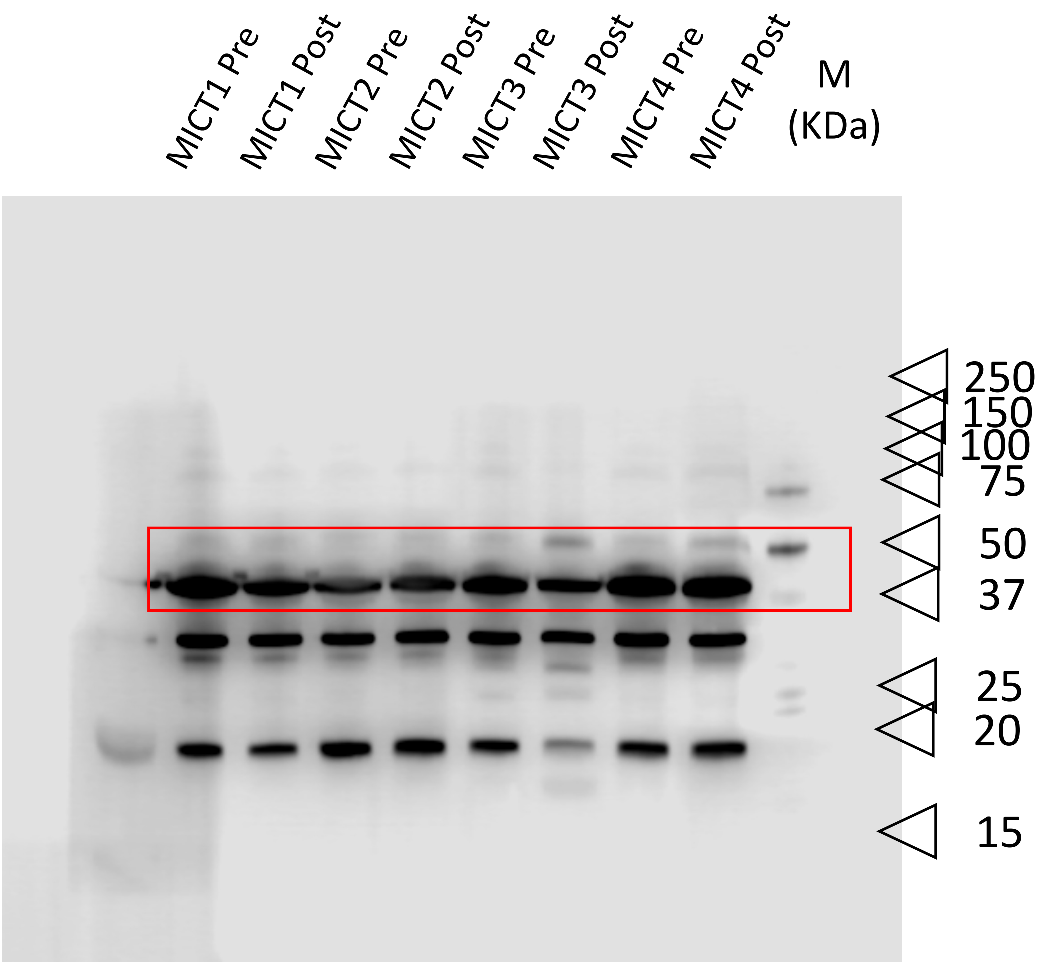

Actin in Red Ponceau

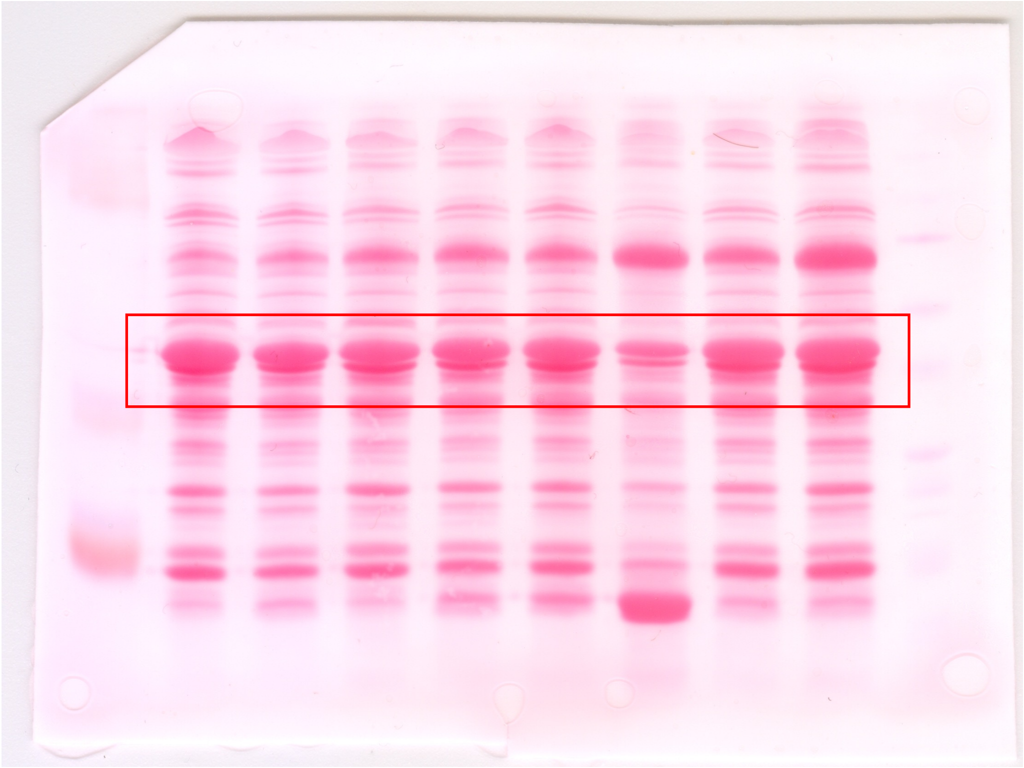

Notes: In figure 5 for MICT, samples are numbered in the order showed in the figure, here are numbered as correspondent to the same subject in agreement with all figures.

PLIN5

PLIN5 HIIT

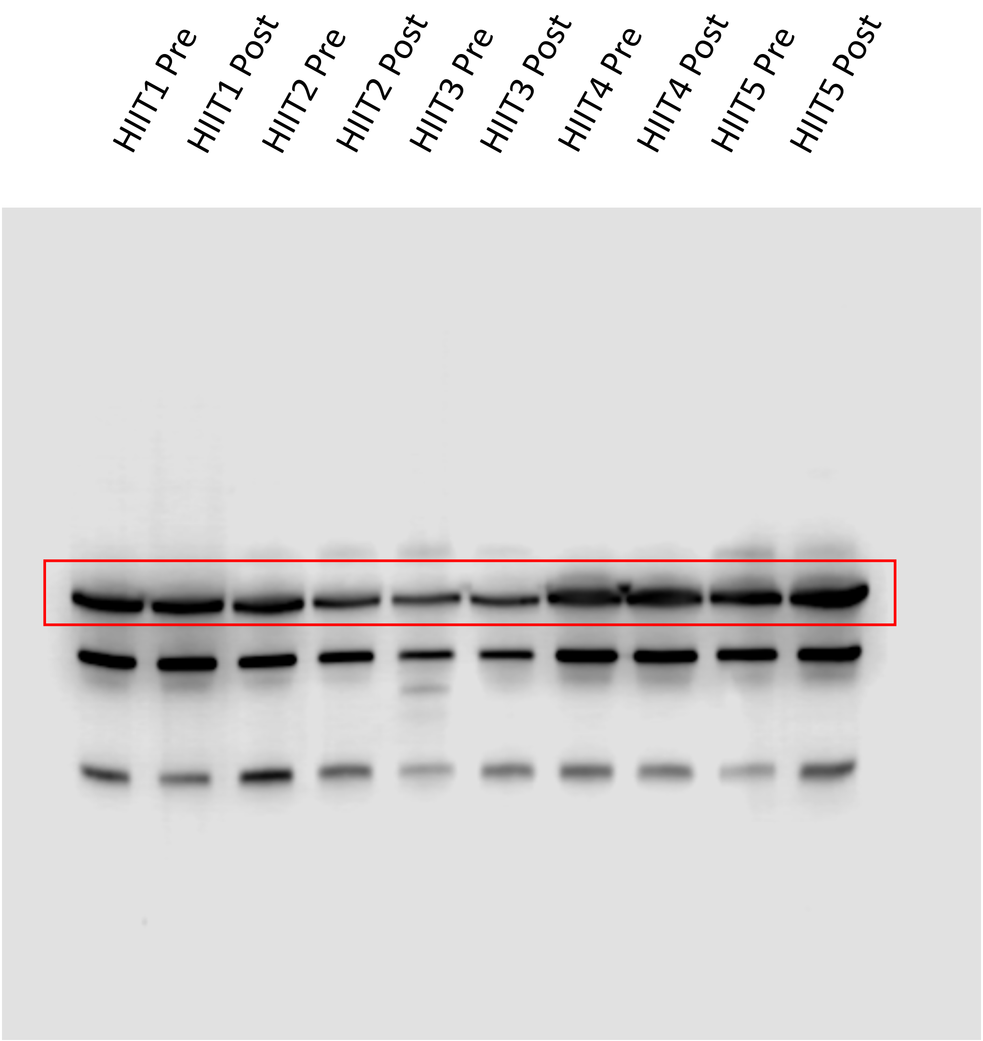

Actin in Red Ponceau

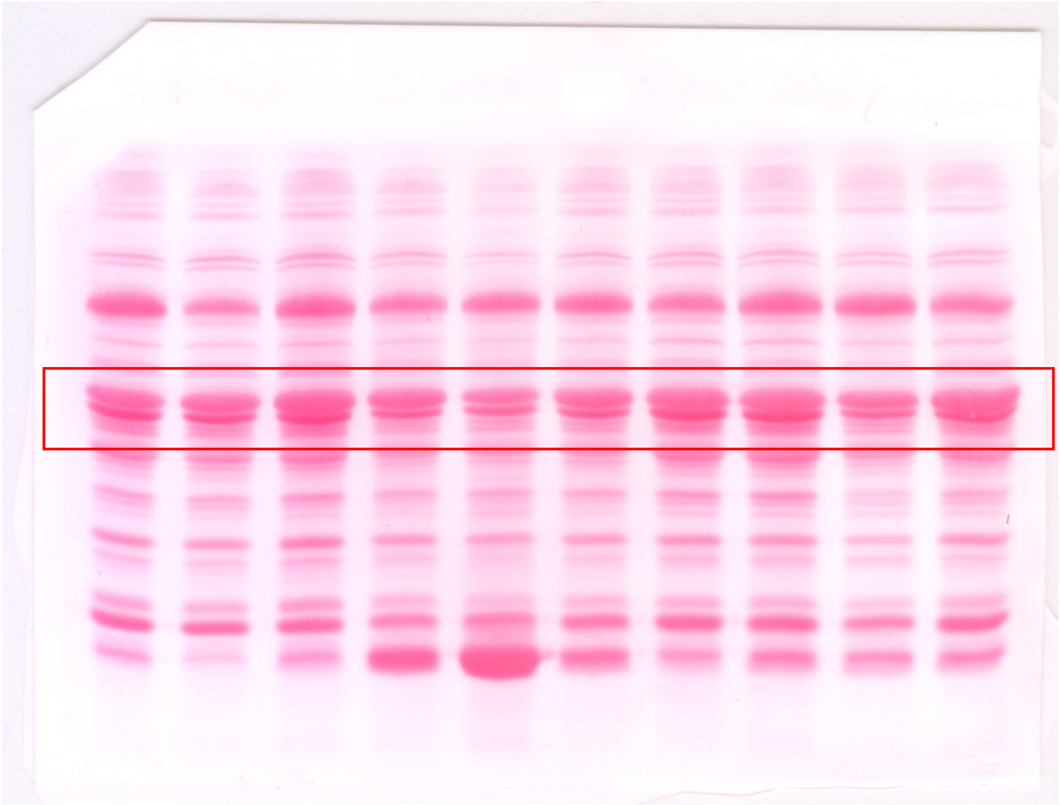

PLIN5

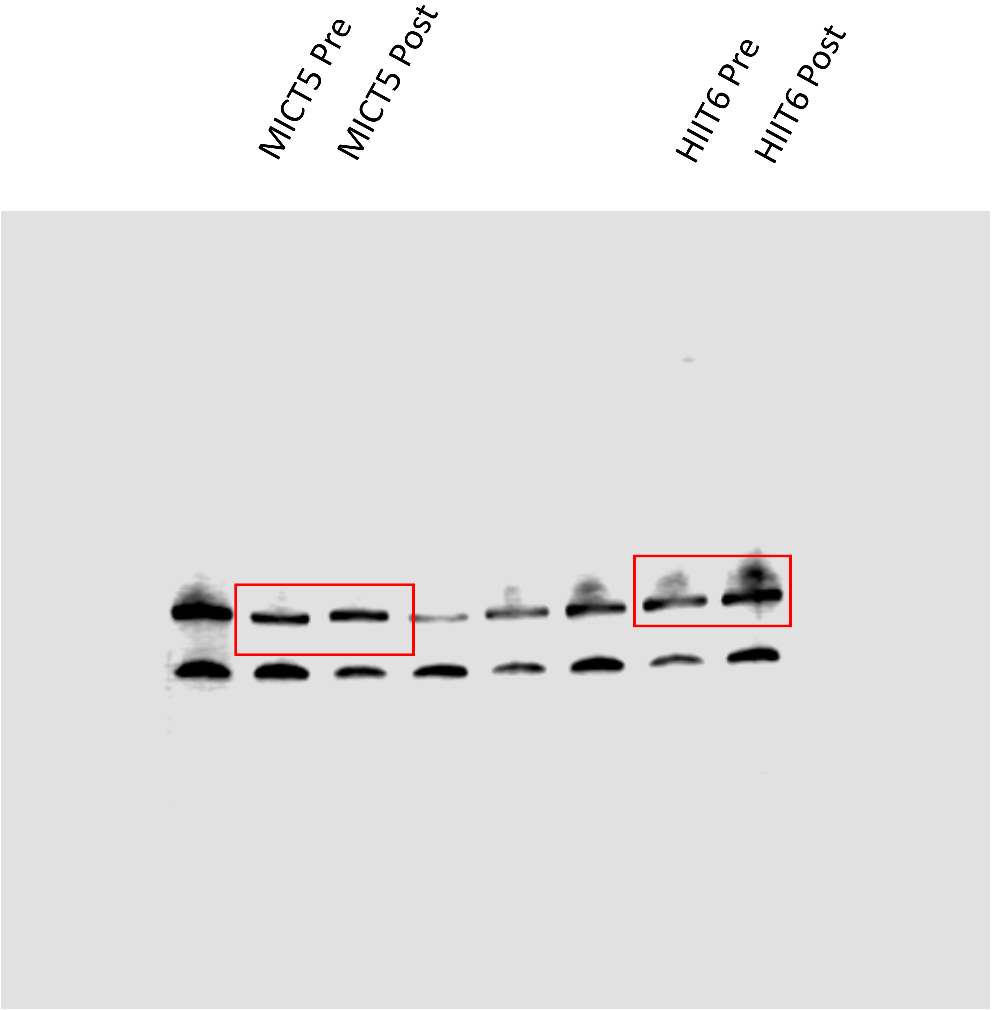

Actin in Red Ponceau

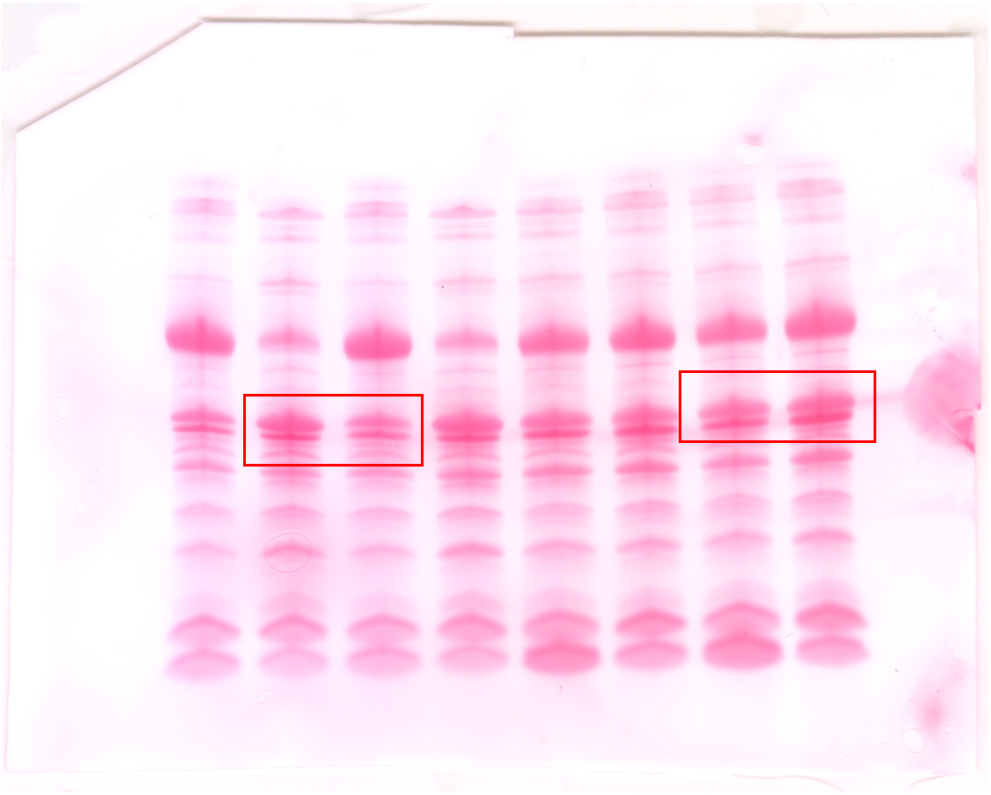

Notes: In figure 5 for MICT, samples are numbered in the order showed in the figure, here are numbered as correspondent to the same subject, in agreement with all figures.
